# Autophagy regulates the effects of ADSC-derived small extracellular vesicles on acute lung injury

**DOI:** 10.1186/s12931-022-02073-y

**Published:** 2022-06-09

**Authors:** Chichi Li, Min Wang, Wangjia Wang, Yuping Li, Dan Zhang

**Affiliations:** 1 Plastic Surgery Department, The First Affiliated Hospital of Wenzhou Medical University, Nanbaixiang, Wenzhou City, Zhejiang Province 325000 People’s Republic of China; 2 Department of Respiratory and Critical Care Medicine, The First Affiliated Hospital of Wenzhou Medical University, Nanbaixiang, Wenzhou City, Zhejiang Province 325000 People’s Republic of China

**Keywords:** Pulmonary microvascular endothelial cells, Lipopolysaccharide, Adipose-derived stem cells, Small extracellular vesicles, Autophagy

## Abstract

Small extracellular vesicles (sEVs) have been recognized to be more effective than direct stem cell differentiation into functional target cells in preventing tissue injury and promoting tissue repair. Our previous study demonstrated the protective effect of adipose-derived stem cells (ADSCs) on lipopolysaccharide (LPS)-induced acute lung injury and the effect of autophagy on ADSC functions, but the role of ADSC-derived sEVs (ADSC-sEVs) and autophagy-mediated regulation of ADSC-sEVs in LPS-induced pulmonary microvascular barrier damage remains unclear. After treatment with sEVs from ADSCs with or without autophagy inhibition, LPS-induced human pulmonary microvascular endothelial cell (HPMVECs) barrier damage was detected. LPS-induced acute lung injury in mice was assessed in vivo after intravenous administration of sEVs from ADSCs with or without autophagy inhibition. The effects of autophagy on the bioactive miRNA components of ADSC-sEVs were assessed after prior inhibition of cell autophagy. We found that ADSC-sEV effectively alleviated LPS-induced apoptosis, tight junction damage and high permeability of PMVECs. Moreover, in vivo administration of ADSC-sEV markedly inhibited LPS-triggered lung injury. However, autophagy inhibition, markedly weakened the therapeutic effect of ADSC-sEVs on LPS-induced PMVECs barrier damage and acute lung injury. In addition, autophagy inhibition, prohibited the expression of five specific miRNAs in ADSC-sEVs -under LPS-induced inflammatory conditions. Our results indicate that ADSC-sEVs protect against LPS-induced pulmonary microvascular barrier damage and acute lung injury. Autophagy is a positive mediator of sEVs function, at least in part through controlling the expression of bioactive miRNAs in sEVs.

**Supplementary Information:**

The online version contains supplementary material available at 10.1186/s12931-022-02073-y.

## Introduction

Sepsis-induced acute lung injury (ALI) is a major cause of acute respiratory distress syndrome, which is a major contributor to high morbidity and mortality. Pulmonary microvascular leakage is one of the characteristics of blood-air barrier dysfunction in ALI [1]. In recent years, multiple experimental and clinical studies have been conducted to clarify the pathogenesis of ALI, and advances have been made in ALI treatment. However, few effective therapies have been developed to improve the outcome of ALI.

Stem cell-related treatments have been shown to be effective in treating injury and the repair of some organs. Adipose-derived stem cells (ADSCs) are a type of mesenchymal stem cell that have been identified as ideal candidates for cell-based therapies based on their relative abundance and easy accessibility [2]. In addition, recent studies have shown that ADSCs have much stronger paracrine potential and tolerance under certain stress conditions than other types of stem cells [3, 4]. Paracrine components, especially exosomes, a type of small extracellular vesicles (sEVs), have been shown to be vital contributors to the efficacy of stem cell paracrine signaling. Exosomes, which are small membraned vesicles (30–100 nm), originate from multivesicular bodies formed by inward budding of the endosomal membrane. Exosomes carry complex biologically active components, including proteins, DNA, mRNA and lipids, among which miRNAs have been suggested to have an effective role in mediating exosome functions [5–7]. However, there is still no method to isolate very pure exosomes, so the term “small extracellular vesicles (sEVs)” but not “exosomes” has been used in present study. Our previous study showed that ADSCs protect against lipopolysaccharide (LPS)-induced pulmonary microvascular barrier damage [8]. However, the effect of ADSC-derived sEVs (ADSC-sEVs) under this condition is still unknown.

Autophagy is a protein and organelle degradation pathway that is pivotal for maintaining cellular homeostasis and promoting survival in response to stress conditions. Recently, the relevance of autophagy to sEVs has been tested. Autophagy affects the production of sEVs, which may be attributed to the link between small extracellular vesicle biogenesis and autophagy via the endolysosomal pathway, and these two processes share common proteins [9–11]. In addition, autophagy is a major cellular degradation process that induces the degradation of various biological molecules, including proteins, lipids, and RNA, some of which are the bioactive components of sEVs [12, 13]. In a previous study, we found that autophagy regulated the release of certain growth factors from ADSCs in LPS-induced lung injury [8]. Based on the aforementioned findings, we hypothesized that autophagy regulates sEV function by regulating the bioactive components in sEVs. The goals of this study were to examine the function of ADSC-sEVs in LPS-induced pulmonary microvascular endothelial barrier injury and determine the role of autophagy in mediating the effects of ADSC-sEVs.

## Materials and methods

### Chemicals and antibodies

LPS (from *Escherichia coli)* (L-2630), FITC-dextran (53379), 3-(4,5-dimethylthiazol-2-yl)-2,5-diphenyltetrazolium bromide (MTT; 11465007001) and a GenElute Mammalian Total RNA Kit (RTN70) were purchased from Sigma-Aldrich (Burlington, MA, USA). Endothelial cell growth medium (1001) was purchased from ScienCell (Carlsbad, CA, USA). A small-interfering RNA construct targeting autophagy-related gene 5 (siATG5, sc-41446), a small-interfering RNA construct targeting autophagy-related gene 7 (siATG7, sc-41447), an siRNA transfection reagent system (sc-41447), an antibody against ATG5 (sc-133158) and an antibody against ATG7 (sc-376212) were purchased from Santa Cruz Biotechnology (Dallas, TX, USA). Anti-microtubule-associated protein 1-light chain 3 (LC3) B (3868), anti-Beclin-1 (3495), anti-p62 (88588S), anti-GAPDH (2118) and anti-rabbit IgG (7074) antibodies were purchased from Cell Signaling Technology (Beverly, MA, USA). EGFP-LC3 plasmid (11546) was bought from Clontech (Mountain View, CA, USA). Lipofectamine™ 3000 transfection reagent (L3000015) was purchased from Thermo Fisher Scientific (Carlsbad, CA, USA). GenElute Mammalian Total RNA Kit (RTN10) was purchased from Sigma-Aldrich (Sigma, St. Louis, MO, USA), A PrimeScript reverse transcription reagent kit with gDNA eraser (RR047A) and One Step PrimeScript™ RT-PCR Kit (RR600A) were purchased from Takara Bio (Kusatsu, Japan). Anti-tumor susceptibility gene (TSG) 101 (ab125011), anti-CD9 (ab92726), anti-CD68 (ab125212), anti- B-cell lymphoma (Bcl)-2 (ab182858), anti-Bcl-2 associated X apoptosis regulator (Bax) (ab32503), anti-zonula occludens-1 (ZO-1) and anti-claudin-5 (ab216880 and ab131259) antibodies were purchased from Abcam (Cambridge, CB2 0AX, UK). Anti-TLR4 (38519S), anti-MyD88 (4283S), anti-NF-κB (8242S) and anti-phosphorylated (p)-NF-κB (3033S) were purchased from Cell Signaling Technology (Danvers, MA, USA). The LIVE/DEAD viability/cytotoxicity kit (L-3224) was purchased from Life Technologies (Carlsbad, CA, USA). Rhodamine-conjugated phalloidin (R415) was purchased from Invitrogen (Carlsbad, NM, USA). A small extracellular vesicle concentration solution kit (UR52111) and small extracellular vesicle purification filter (UR90102) were purchased from Umibio (Shanghai, China). Micro BCA protein assay kit (ZY80816) was purchased from Zeye Biotechnology (Shanghai, China). A mouse total protein S (TPS) ELISA Kit (BPE20886) was purchased from Lengton Bioscience (Shanghai, China). Enzyme-linked immunosorbent assay (ELISA) kits for tumor necrosis factor (TNF)-α (EM3184S) and interleukin (IL)-1β (EM3184S) were purchased from Biotech Well (Shanghai, China).

### Adipose-derived stem cell culture and treatment

Human ADSCs, purchased from Cyagen Biosciences (Santa Clara, CA, USA), were cultured in DMEM. The primary cells were harvested when they had grown to approximately 80% confluence, and then the cells were plated on new culture dishes at approximately 6000 cells/cm^2^. To determine whether autophagy influenced ADSC-sEV effects on LPS-induced microvascular barrier damage, we constructed ADSC lines with or without autophagy inhibition using an siRNA targeting ATG5. For siRNA transfection, 2 × 10^6^ cells were transfected with 50 nM siATG5 using an siRNA transfection reagent system. After 36 h, the autophagy level of the cells was measured. Then, the cells were treated with interleukin (IL)-1β for 6 h, and sEVs were collected according to the undermentioned experimental method. ADSC^siRNA−NC^-sEVs represent sEVs derived from ADSCs transfected with negative control siRNA. ADSC^siATG5^-sEVs and ADSC-sEVs represent sEVs derived from ADSCs with and without autophagy inhibition, respectively.

### Isolation of sEVs

For small extracellular vesicle isolation, small extracellular vesicle concentration solution was used. Briefly, ADSCs were washed with PBS several times and cultured in DMEM supplemented with 10% exosome-free fetal bovine serum. After reaching confluence, the cells were treated with DMEM containing 1 ng/ml recombinant human IL-1β and incubated for 24 h. The culture medium was collected and centrifuged at 3000×*g* for 15 min at 4 °C, followed by centrifugation at 2500×*g* for 30 min. The supernatant was then filtered and mixed with small extracellular vesicle concentration solution at a 4:1 ratio, and the mixture was mixed well with a vortex oscillator for 1 min, and set at 4 °C for 2 h, and then was high-speed centrifuged at 10,000×*g* for 1 h at 4 °C. Then, the pellets were then resuspended in the 1 × PBS at a 100:1 ratio and high-speed centrifuged at 12,000×*g* for 2 min at 4 °C. Finally, the supernatant containing extracted sEVs were collected and transferred to the upper chamber of sEVs purification filter, centrifuged at 3000×*g* for 10 min at 4 °C, then the liquid at the bottom of the sEVs purification filter was collected.

### Human pulmonary microvascular endothelial cell culture and in vitro cell groupings

Human pulmonary microvascular endothelial cells (PMVECs) (PromoCell, Heidelberg, Germany) were cultured in an endothelial cell medium. The cells were detached and transferred to new dishes at a split ratio of 1:2 for further propagation until they grew to confluence (usually 3–5 day). PMVECs at passages 3–5 were selected for analysis. PMVECs were divided into four groups as follows: PMVECs, LPS-challenged PMVECs, and LPS-challenged PMVECs cultured with ADSC-sEVs or ADSC^siATG5^-sEVs. To mimic LPS-induced lung microvascular injury, PMVECs were incubated in endothelial cell medium supplemented with 10% fetal bovine serum containing 100 ng/ml LPS, followed by the addition of 20 μg/ml sEVs in 100 μl of PBS. The doses of sEVs were determined according to previous studies with minor modifications [14, 15]. After 24 h, the cells were collected for further study.

### EGFP-LC3 plasmid transfection

EGFP-LC3 plasmids were transfected with Lipofectamine™ 3000 transfection reagent according to the manufacturer’s protocol. After transfection for 48 h, the EGFP-LC3 puncta fluorescent signal were visualized using a Leica TCS SP8 confocal microscope and counted in × 100 fields (five sequential fields were counted and averaged per coverslip) for three coverslips.

### Real-time reverse transcription polymerase chain reaction (RT-PCR)

RT-PCR was performed to determine relative mRNA levels of LC3, Beclin-1 and p62 in ADSCs. Total RNA was isolated from ADSCs by using a GenElute Mammalian Total RNA Kit according to the manual. The purified RNA was used for reverse transcription using One Step RT-PCR. Primers used in the PCR reaction were as Table 1. Thecycle conditions were: 48 °C for 30 min and 94 °C for 1 min, followed by 40 cycles at 94 °C for 15 s, 65 °C for 30 s and at 68 °C for 1 min. Polymerase chain reaction products were detected with 2% agarose gel electrophoresis and analyzed with a gel imaging analyzer. The ratio of the target gene (LC3, Beclin-1 and p62) OD to the reference gene (β-Actin) OD in the same sample was calculated and considered as the target gene mRNA relative content. All the assays were performed in duplicate.

**Table 1:** List of primers for RT-PCR

Target name	Sequence	Tm (℃)
LC3	F: CCTGGACAAGACCAAGTT	48.3
	R: GAGGCGTAGACCATATAGAG	48.7
Beclin-1	F: GCTGCCGTTATACTGTTCT	52.7
	R: CCTCCTGTGTCTTCAATCTT	57.7
P62	F: ATGGAGTCGGATAACTGTTC	50.9
	R: GGATTCTGGCATCTGTAGG	50.1
β-Actin	F: CATCCGCAAAGACCTGTACG	65.3
	R: CCTGCTTGCTGATCCACATC	65.2

### Identification and quantification of ADSC-derived sEVs

According to previous reports [16, 17], transmission electron microscopy was used to observe the double-layer ultrastructure of purified ADSC-sEVs. Nanoparticle tracking analysis was used to determine the average diameter and concentration of sEVs. Proteins from ADSC-sEVs were extracted by RIPA lysis buffer. The common sEV-specific protein markers TSG101, CD9 and the nonspecific protein CD68 were measured via western blotting as the following procedures. Quantification of sEVs protein concentration was detected by Micro BCA Protein Assay Kit according to the manufacturer’s instructions.

### Protein preparation and immunoblotting

ADSCs or PMVECs were homogenized in RIPA lysis buffer, and then the homogenate was incubated on ice for 45 min and centrifuged at 4 °C (12,000 g for 5 min). After determining the protein concentration, the protein was collected and separated via sodium dodecyl sulfate polyacrylamide gel electrophoresis at 120 V for 2 h. The proteins in the gels were transferred onto a polyvinylidene difluoride membrane, which was then incubated with specific primary antibodies, followed by incubation with horseradish peroxidase-conjugated secondary antibody for 1 h. Finally, protein visualization was performed using Pierce ECL western blotting substrate and autoradiography. The following primary antibodies were used: anti-LC3B, anti-Beclin-1, anti-p62, anti-ATG5, anti-ZO-1, anti-claudin-5, anti-TSG101, anti-CD9, anti-CD68, anti-Bcl-2, anti-Bax, anti-TLR4, anti-MyD88, anti-NF-κB and anti-p-NF-κB and anti-GAPDH. Quantity One 4.6 software was used to analyze the blots. The data were normalized to GAPDH and are expressed as the optical density (OD) integration.

### Trans-endothelial permeability assay

PMVECs were cultured on the upper wells in a Transwell system, and FITC-dextran (1 mg/ml, MW 40,000) was added to the top of the wells and allowed to permeate through the PMVEC monolayer. After LPS treatment and ADSC-sEV culture for 6 h, the medium was collected from the lower compartments of the Transwell chambers and replaced with an equal volume of basal cell medium. The fluorescence value of FITC-dextran in the medium was determined with a fluorescence microplate reader (FLX800TBID, BioTek Instruments, Inc., Winooski, VT, USA) at an excitation wavelength of 492 nm and an emission wavelength of 520 nm.

### Detection of PMVEC viability

We used an MTT assay to assess the viability of PMVECs. Each group was analyzed in triplicate at a density of 2000 cells/well. The cells were incubated with 5 mg/ml MTT during the last 4 h of LPS challenge. After removal of the supernatant, 100 ml of dimethyl sulfoxide was added to each well, followed by 10 min of shaking to dissolve the crystals. The OD of each well was measured at 490 nm with a spectrophotometer. The experiment was repeated three times in each group.

A LIVE/DEAD viability/cytotoxicity kit was used to further measure cell viability. Briefly, the cells were cultured on sterile glass coverslips as confluent monolayers. Then, 20 μl of 2 mM ethidium homodimer (EthD)-1 was added to 10 ml of PBS and combined with 5 μl of a 4 mM calcein AM solution. The working solution, which contained 2 μM calcein AM and 4 μM EthD-1, was directly added to the cells. After 15 min, the cells were examined using a confocal laser-scanning microscope.

### Detection of apoptosis via flow cytometry

An Annexin V-FITC apoptosis detection kit and flow cytometry were used to determine the apoptosis rate according to the manufacturer’s instructions. Briefly, PMVECs were digested with 0.25% trypsin and then rinsed twice with PBS. Then, the cells were resuspended in 1 × binding buffer at a concentration of 1 × 10^6^ cells/ml, and 100 µl of the resuspended cell solution was transferred to 5-ml culture tubes. Then, 5 µl of Annexin V-FITC and 5 µl of propidine iodide were added to the culture tubes. The resulting solution was incubated at room temperature in the dark for 15 min, after which 400 µl of 1 × binding buffer was added. The apoptosis rates were analyzed immediately via flow cytometry (BD Biosciences, San Jose, CA, USA).

### F-actin labeling

We determined stress fiber formation by measuring F-actin using a rhodamine-conjugated phalloidin molecular probe according to the manufacturer’s instructions. Cells were treated with 100 ng/ml LPS and ADSC-sEVs, fixed with 3.7% paraformaldehyde for 10 min, permeabilized with 0.5% Triton X-100, and finally stained with rhodamine-conjugated phalloidin. The nuclei were labeled with 4′,6-diamidino-2-phenylindole. The labeled cells were analyzed under a Nikon A1 R laser confocal microscope. We quantified F-actin levels in different groups by analyzing the percentage of cells containing stress fibers.

### Establishment of LPS-induced acute lung injury mouse models and study grouping

Eight- to ten-week-old wild-type BALB/c mice (Animal Center, Wenzhou medical University, Wenzhou, China) were used. The mice were starved of solid food but had free access to water 12 h before the experiments. All experimental protocols were approved by the Animal Care Ethics Committee of Wenzhou Medical University. All mice were handled in compliance with the Guidelines for the Care and Use of Laboratory Animals [18]. The mice were anesthetized with pentobarbital sodium (50 mg/kg intraperitoneally), orally intubated with a sterile plastic catheter and challenged by intratracheal instillation of 2 mg of LPS kg^−1^ b.w. ADSC-sEVs or ADSC^siATG5^-sEVs (100 μg/ml, 200 μl total volume) were intravenously administered via a caudal venous canula 30 min following LPS challenge as described previously [15]. The mice were sacrificed 48 h after LPS instillation.

### Lung histopathology and edema detection

The mice were sacrificed 48 h after LPS instillation, and fresh left lung tissue was dissected and fixed immediately in 10% formalin and then paraffin-embedded and cut into 4-μm thick paraffin sections. The sections were stained with hematoxylin and eosin (H&E). Lung injury was assessed according to four categories: interstitial inflammation, neutrophil infiltration, congestion, and edema. The assessment results are shown as scores on a 0- to 4-point scale: no injury = score of 0; injury in 25% of the field = score of 1; injury in 50% of the field = score of 2; injury in 75% of the field = score of 3; and injury throughout the field = score of 4 [19]. Ten microscopic fields from each slide were analyzed. The sums of the tissue slides were averaged to evaluate the severity of lung injury. All microscopic sections were scored by a pathologist who was blinded to the experimental groups and protocol.

In addition, the right lungs of the mice were immediately removed, and the wet weight was measured after the experiment. Then, the same lungs were dried at 56 °C for 72 h. The wet/dry lung weight ratio was then calculated to assess the severity of lung edema.

### Protein concentration in bronchoalveolar lavage fluid (BALF)

After the experiment, the left general bronchus of each mouse was ligated, and then the right lung was irrigated with 0.5 ml PBS after LPS challenge. The fluid from three lavages was pooled. The total BALF from each mouse was centrifuged at 4 °C (1000 rpm for 15 min). The protein concentration of the BALF supernatants was determined using a mouse total protein S Kit according to the manufacturer’s instructions.

### Detection of cytokines via ELISA

The expression of the inflammatory cytokine TNF-α and IL-1β in the BALF was measured using ELISA kits in accordance with the manufacturer’s instructions.

### Quantification of five specific miRNAs in sEVs using real-time RT-PCR

Total RNA was isolated from sEVs using a GenElute Mammalian Total RNA Kit according to the manufacturer’s instructions. cDNA synthesis was executed using a PrimeScript reverse transcription reagent kit with gDNA eraser. Reverse transcription was performed using a One Step PrimeScript™ RT-PCR Kit. The sequences of the forward primers used are shown in Table 2.

**Table 2:** List of forward primers for RT-PCR

Target name	Sequence	Tm (℃)
let-7a-1	CTATACAATCTACTGTCTTTCCAAAAA	47.4
miR-21a	AACAGCAGTCGATGGGC	47.7
miR-143	TGAGATGAAGCACTGTAGCAAA	51.9
miR-145a	ATTCCTGGAAATACTGTTCTTAAAA	55.8
miR-451a	AACCGTTACCATTACTGAGTTAAAA	49.7

### Statistical analysis

Data were obtained from at least three separate experiments performed in triplicate. GraphPad Prism 5 software was used for data processing. The results are shown as the mean ± standard deviation (SD). Differences between groups were determined by one-way analysis of variance and post hoc Bonferroni corrections for multiple comparisons. The histologic semiquantitative analysis was compared using a nonparametric Mann–Whitney test. A *P-value* < 0.05 was considered to be statistically significant.

## Results

### Effective inhibition of autophagy using siATG5 or siATG7

**Fig. 1:**
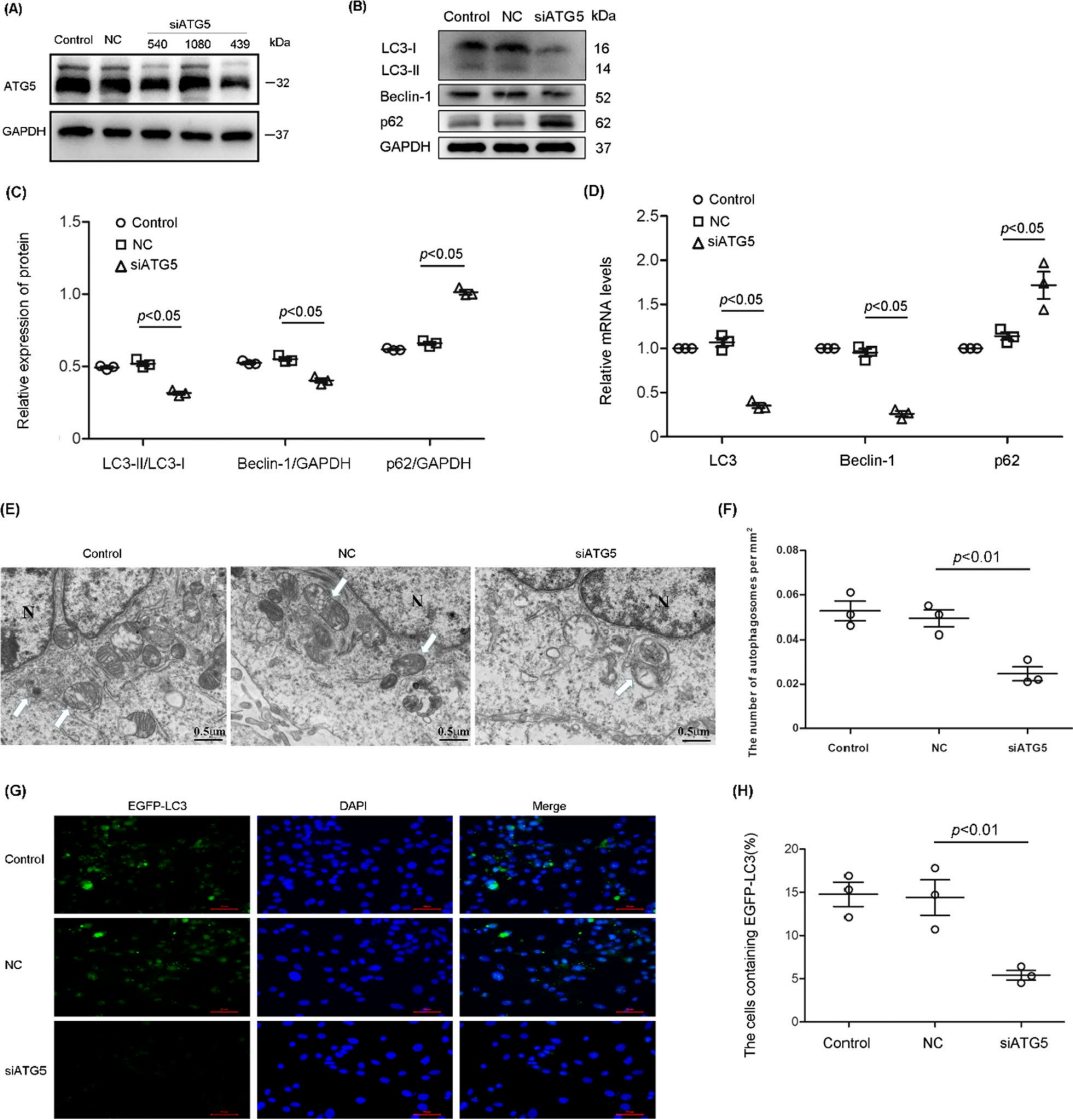
Effects of Atg5-siRNA on autophagy in ADSCs. **A, B** Representative western blots showing expression of ATG5, LC3, Beclin-1 and p62 in ADSCs. The expression of ATG5 was most effectively inhibited in siATG5 439-transfected ADSCs. **C** Statistical analysis of LC3, Beclin-1 and p62 expression. siATG5 treatment markedly inhibited expression of LC3-II and Beclin-1 but promoted that of p62 (the relative expression of LC3-II, control, 0.49 ± 0.01 vs. NC, 0.52 ± 0.03 vs. siATG5, 0.32 ± 0.02. the relative expression of Beclin-1, control, 0.53 ± 40.01 vs. NC, 0.55 ± 0.02 vs. siATG5, 0.40 ± 0.03. The relative expression of p62, Control, 0.62 ± 0.01 vs. NC, 0.66 ± 0.02 vs. siATG5, 1.01 ± 0.03). **D** Relative mRNA level of LC3, Beclin-1 and p62. siATG5 treatment decreased the mRNA level of LC3 and Beclin-1, but increased that of p62. (the relative mRNA level of LC3, control, 1.00 ± 0.00 vs. NC, 1.07 ± 0.09 vs. siATG5, 0.35 ± 0.05. the relative mRNA level of Beclin-1, control, 1.00 ± 0.00 vs. NC, 0.95 ± 0.08 vs. siATG5, 0.26 ± 0.06. the relative mRNA level of p62, control, 1.00 ± 0.00 vs. NC, 1.14 ± 0.08 vs. siATG5, 1.72 ± 0.27). **E** Transmission electron microscopy images showing characteristic autophagic ultrastructure in the cells. Autophagosomes are indicated by white arrows. **F** Quantitative analysis of the number of autophagosomes per mm^2^ of cell cross sections. The number of autophagosomes per mm^2^ of cell cross section in the siATG5-treated group was significantly lower than that in the control group (the number of autophagosomes per mm^2^ of cell cross section, control, 0.053 ± 0.008 vs. NC, 0.049 ± 0.007 vs. siATG5, 0.025 ± 0.006). **G** Fluorescent images of EGFP-LC3 in ADSCs. **H** Statistical analysis of the number of cells containing LC3-positive puncta in different groups. The number of cells containing LC3-positive puncta in LPS treatment group was much greater than that in control group, and the number of puncta was decreased by siATG5 treatment (the number of cells containing LC3-positive puncta (%), control, 14.77 ± 2.44 vs. NC, 14.40 ± 3.56 vs. siATG5, 5.40 ± 0.95). The results are expressed as the mean ± SD of three independent experiments. ADSC-NC represents ADSCs transfected with negative control siRNA. ADSC-siATG5 represents ADSC transfected with siATG5

ATG5 is indispensable in both canonical and noncanonical autophagy. Through siATG5 treatment, we effectively reduced autophagy levels. Western blotting demonstrated that the expression of ATG5 was most effectively diminished in siATG5 439-transfected ADSCs (Fig. 1A), and thus, siATG5 439 was selected to inhibit autophagy in subsequent experiments. siATG5 treatment significantly lowered the expression of ATG5, LC3-II and Beclin-1, two essential autophagy-related proteins, but increased the expression of p62, an autophagy substrate (Fig. 1B, C). The changes trend of LC3, Beclin-1 and p62 mRNAs was similar to that of the corresponding proteins (Fig. 1D). Morphological assessment via transmission electron microscopy showed that autophagosomes were double- or multimembrane structures that engulfed cytoplasmic components (Fig. 1E). Statistically, the number of autophagosomes per mm^2^ of cell cross section in the siATG5-treated group was significantly lower than that in the control group (Fig. 1F). In another experiment, we examined the autophagosome structures by EGFP-LC3 immunostaining (Fig. 1G). The number of cells containing LC3-positive puncta in LPS treatment group was much greater than that in control group, and the number of puncta was decreased by siATG5 treatment (Fig. 1H). For further testifying the role of autophagy in mediating effects of ADSC-sEV on LPS-induced lung injury, we inhibited the expression of ATG7, another essential protein in autophagy process. As shown, siATG7 treatment effectively inhibited the expressions of ATG7, LC3-II and Beclin-1, but promoted that of p62 significantly (Additional file 1: Fig. S1).

### Isolation and characterization of ADSC-derived sEVs

**Fig. 2:**
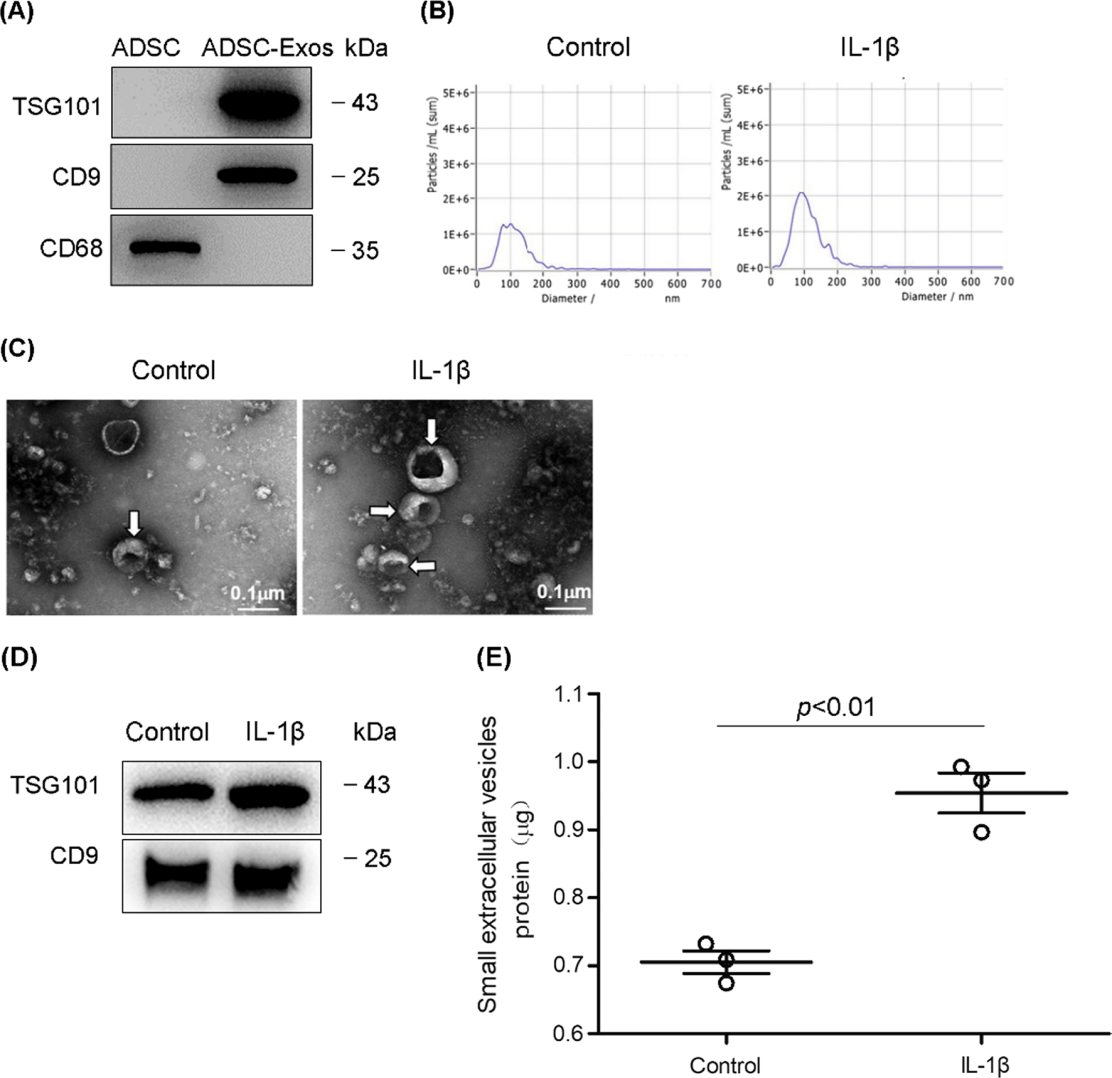
Characteristics of ADSC-sEVs. **A** Representative western blot analysis of small extracellular vesicles (sEVs) showing the presence of TSG 101 and CD9 but the absence of CD68 in ADSC-sEVs. **B** Nanoparticle tracking analysis of sEVs shows a single peak at 100 nm, and IL-1β preconditioning induced more sEVs production. **C** Electron microscopy showing the cup-shaped morphology of sEVs in both control and IL-1β-preconditioned ADSCs. **D** Representative western blot analysis of the expressions of TSG 101 and CD9 in ADSC-sEVs in presence or absence of IL-1β preconditioning. **E** Quantitative analysis of total amount protein in ADSC-sEVs in presence or absence of IL-1β preconditioning. IL-1β preconditioning promoted the production of these extracellular vesicles in ADSCs (quantification of the proteins in ADSC-sEVs (μg), control, 0.71 ± 0.03 vs. IL-1β, 0.95 ± 0.05). The results are expressed as the mean ± SD of three independent experiments

Previous studies have shown that preconditioning mesenchymal stem cells with cytokines or specific conditioned medium can enhance their paracrine functions, including the effects of exosomes on tissue injury and repair [20–22]. In this study, we preconditioned ADSCs for 6 h with IL-1β, one of the vital proinflammatory cytokines induced by LPS, and then collected ADSC-derived extracellular vesicles using a small extracellular vesicles concentration solution kit and a small extracellular vesicle purification filter. Western blotting demonstrated the presence of the small extracellular vesicle marker proteins TSG101 and CD9 but the absence of CD68 in these vesicles (Fig. 2A). In addition, the isolated ADSC-derived extracellular vesicles ranged in size from 70 to 120 nm, as determined by nanoparticle tracking analysis (Fig. 2B). Transmission electron microscopy analysis showed that isolated ADSC-derived extracellular vesicles had a typical cup-shaped morphology in both the control and IL-1β preconditioning groups (Fig. 2C). Western blotting demonstrated the presence of the small extracellular vesicle marker proteins TSG101 and CD9 in ADSC-sEVs preconditioned with IL-1β (Fig. 2D). IL-1β preconditioning promoted the production of these extracellular vesicles as shown by the quantitative analysis of sEVs total protein (Fig. 2E). These findings indicated that these vesicles fulfilled the minimal experimental criteria for sEVs [17]. Therefore, these vesicles are referred to as ADSC-small extracellular vesicles (sEVs).

In the following experiment, we compared the effect of ADSC-sEVs with or without IL-1β preconditioning on LPS-induced pulmonary microvascular injury. We found ADSC-sEVs with IL-1β preconditioning have much better protective effect on alleviating LPS-induced PMVECs apoptosis than that from ADSCs without IL-1β preconditioning (Additional file 1: Fig. S2). However, in vivo experiment, the protective effect of IL-1β preconditioned ADSC-sEVs on LPS-induced acute lung injury was similar to that of ADSC-sEVs without IL-1β preconditioning (Additional file 1: Fig. S3). We collected ADSC-sEVs from IL-1β-preconditioned ADSCs for further experiments.

### Autophagy inhibition reduced the protective effect of ADSC-sEVs on the expression of tight junction-related proteins

For detecting the effects of autophagy on the protective effect of ADSC-sEVs on LPS-induced lung injury, we firstly examined whether inhibiting autophagy with siATG5 would affect the physiological characteristic of ADSC-sEVs. As shown, siATG5 treatment did not affect the physiological characteristic of ADSCs-sEVs (Additional file 1: Fig. S4). To further test the effect of autophagy on ADSC-sEVs function in LPS-induced pulmonary microvascular barrier damage, we extracted equal quantity of sEVs from ADSCs in the presence or absence of autophagy inhibition as shown by the quantification of total protein from ADSC-sEVs (Additional file 1: Fig. S5).

**Fig. 3:**
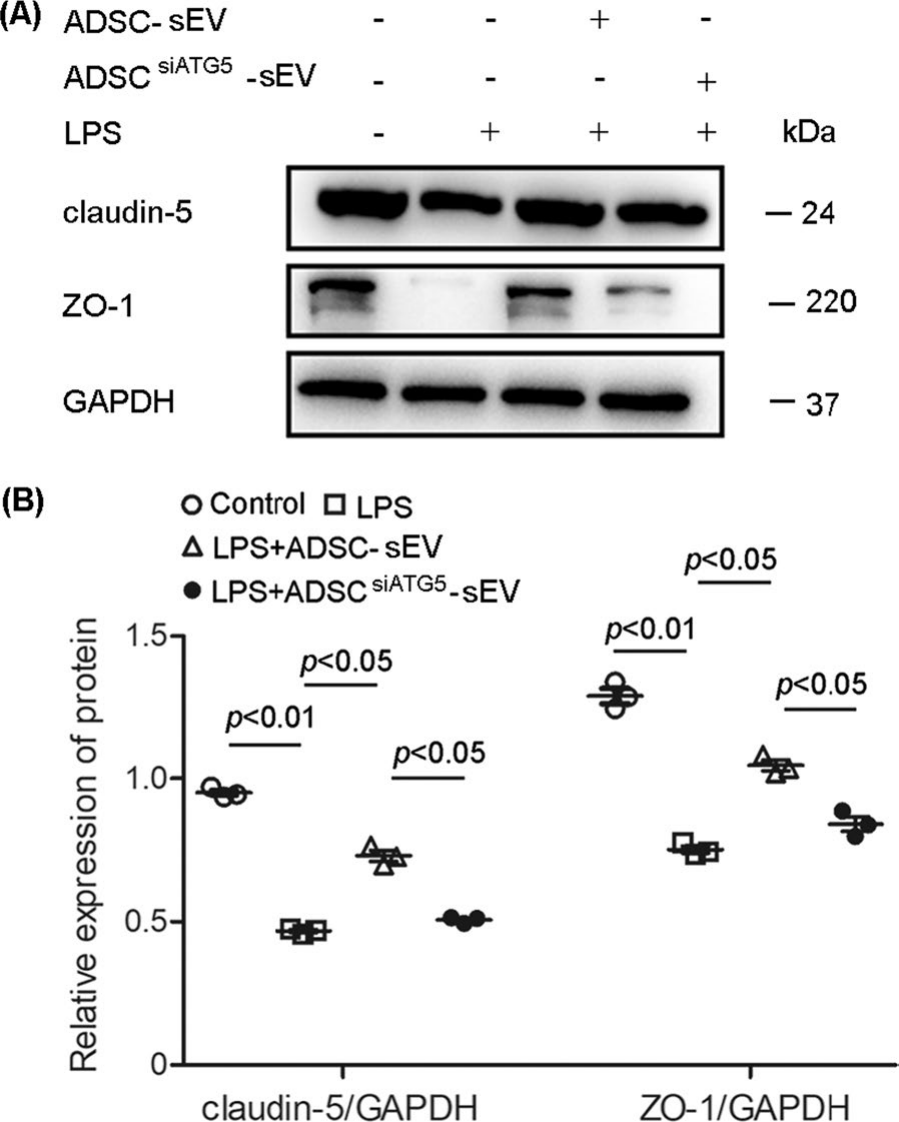
Autophagy mediated the effects of ADSC-sEVs on tight junction-associated protein expression in LPS-treated PMVECs. **A** Representative western blots showing ZO-1 and claudin-5 expression in PMVECs. **B** Statistical analysis of ZO-1 and claudin-5 expression in PMVECs. LPS decreased the expression of ZO-1 and claudin-5 in PMVECs, which was significantly inhibited by ADSC-sEVs treatment. Autophagy inhibition weakened the effect of ADSC-sEVs on the expression of ZO-1 and claudin-5 (the relative expression of claudin-5, control, 0.95 ± 0.02 vs. LPS, 0.47 ± 0.01 vs. LPS + ADSC-sEV, 0.73 ± 0.03, LPS + ADSC^siATG5^-sEV, 0.51 ± 0.01. the relative expression of ZO-1, control, 1.29 ± 0.05 vs. LPS, 0.75 ± 0.02 LPS + ADSC-sEV, 1.05 ± 0.03 vs. LPS + ADSC^siATG5^-sEV 0.84 ± 0.04). The results are expressed as the mean ± SD of three independent experiments

ADSC-sEVs were added to PMVECs in the presence of LPS. We found that LPS inhibited the expression of ZO-1 and claudin-5, two critical tight junction-related proteins in PMVECs. However, ADSC-sEV treatment, significantly inhibited this change in PMVECs, and autophagy inhibition weakened the effect of ADSC-sEVs on the expression of ZO-1 and claudin-5 (Fig. 3A, B).

### Autophagy inhibition reduced the protective effect of ADSC- sEVs on PMVEC apoptosis and viability

**Fig. 4:**
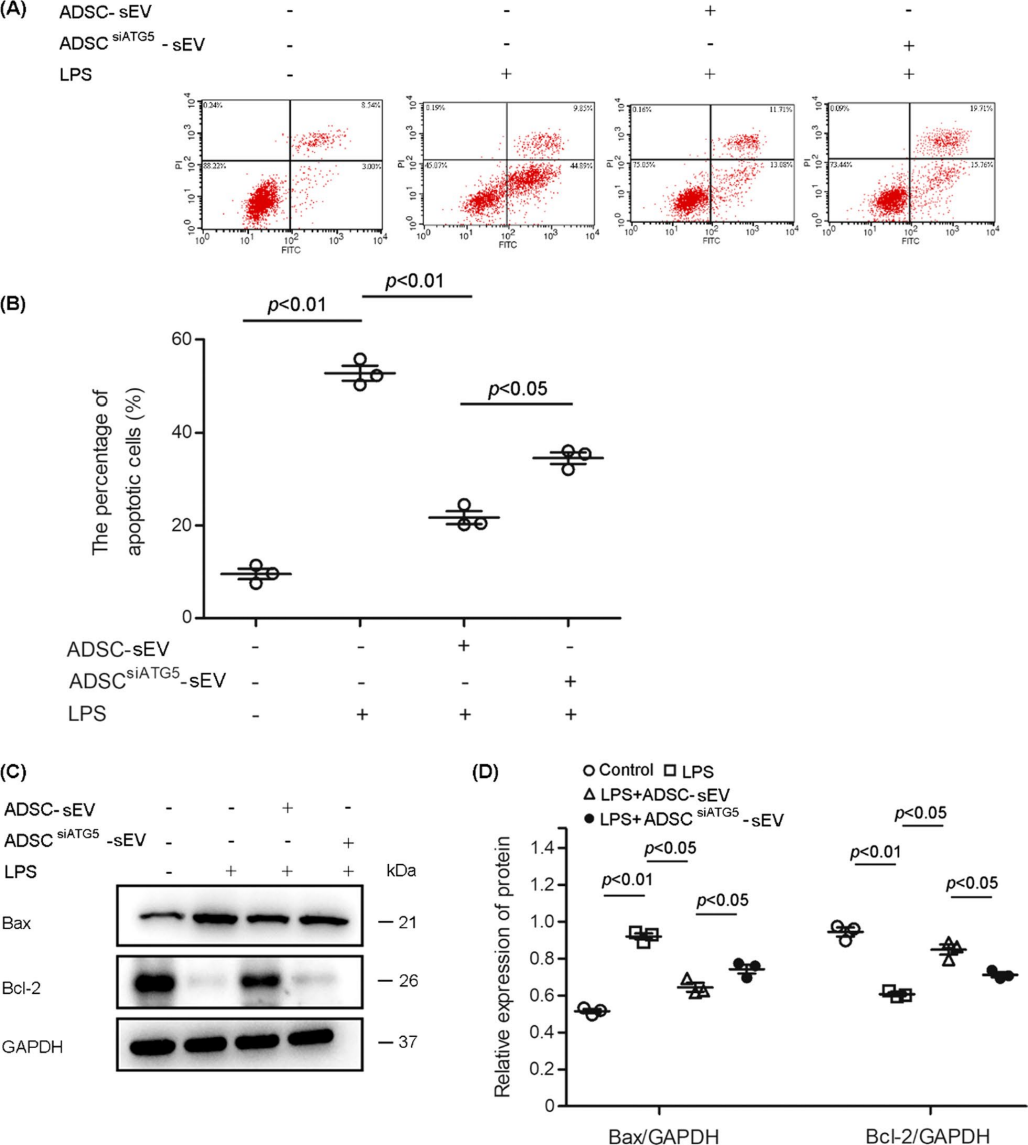
Autophagy inhibition weakened the inhibitory effect of ADSC-sEVs on LPS-induced PMVEC apoptosis and viability. **A**, **B** Typical flow cytometry quadrant diagrams and corresponding statistical analysis of apoptotic PMVECs. The top left, top right, and bottom right plots represent necrotic cells and late and early apoptotic cells, respectively. LPS markedly increased the percentage of endothelial cell apoptosis, which was effectively reduced by ADSC- sEVs. However, autophagy inhibition, significantly weakened the function of ADSC-sEVs (the percentage of endothelial cell apoptosis (%), control, 9.55 ± 1.96 vs. LPS, 52.73 ± 2.79 vs. LPS + ADSC-sEV, 21.73 ± 2.43 vs. LPS + ADSC^siATG5^-sEV 34.57 ± 2.15). **C**, **D** Representative western blots and statistical analysis of Bax and Bcl-2 expression in PMVECs. LPS promoted the expression of Bax and reduced the expression of Bcl-2, ADSC- sEVs treatment inhibited the expression of Bax and promoted that of Bcl-2 under LPS stimulation. Autophagy inhibition weakened these effects of ADSC-sEVs (the relative expression of Bax, control, 0.52 ± 0.02 vs. LPS, 0.92 ± 0.03 vs. LPS + ADSC-sEV, 0.64 ± 0.04 vs. LPS + ADSC^siATG5^-sEV, 0.74 ± 0.04. the relative expression of Bcl-2, control, 0.94 ± 0.04 vs. 0.61 ± 0.02 vs. LPS + ADSC-sEV, 0.85 ± 0.05 vs. LPS + ADSC^siATG5^-sEV, 0.71 ± 0.02). The results are expressed as the mean ± SD of three independent experiments

PMVEC apoptosis has been used as one of the critical assessment indices for LPS-induced pulmonary microvascular barrier damage [23]. Flow cytometry showed that LPS markedly increased the percentage of endothelial cell apoptosis, which was effectively reduced by ADSC- sEVs. However, autophagy inhibition by siATG5 or siATG7, significantly weakened the function of ADSC-sEVs (Fig. 4A, B and Additional file 1: Fig. S6). In addition, we measured the expression of Bax and Bcl-2, which are classic pro- and antiapoptotic proteins, respectively. LPS promoted the expression of Bax and reduced the expression of Bcl-2; ADSC- sEVs inhibited the expression of Bax and promoted that of Bcl-2 under LPS stimulation. Autophagy inhibition weakened these effects of ADSC-sEVs (Fig. 4C, D).

**Fig. 5:**
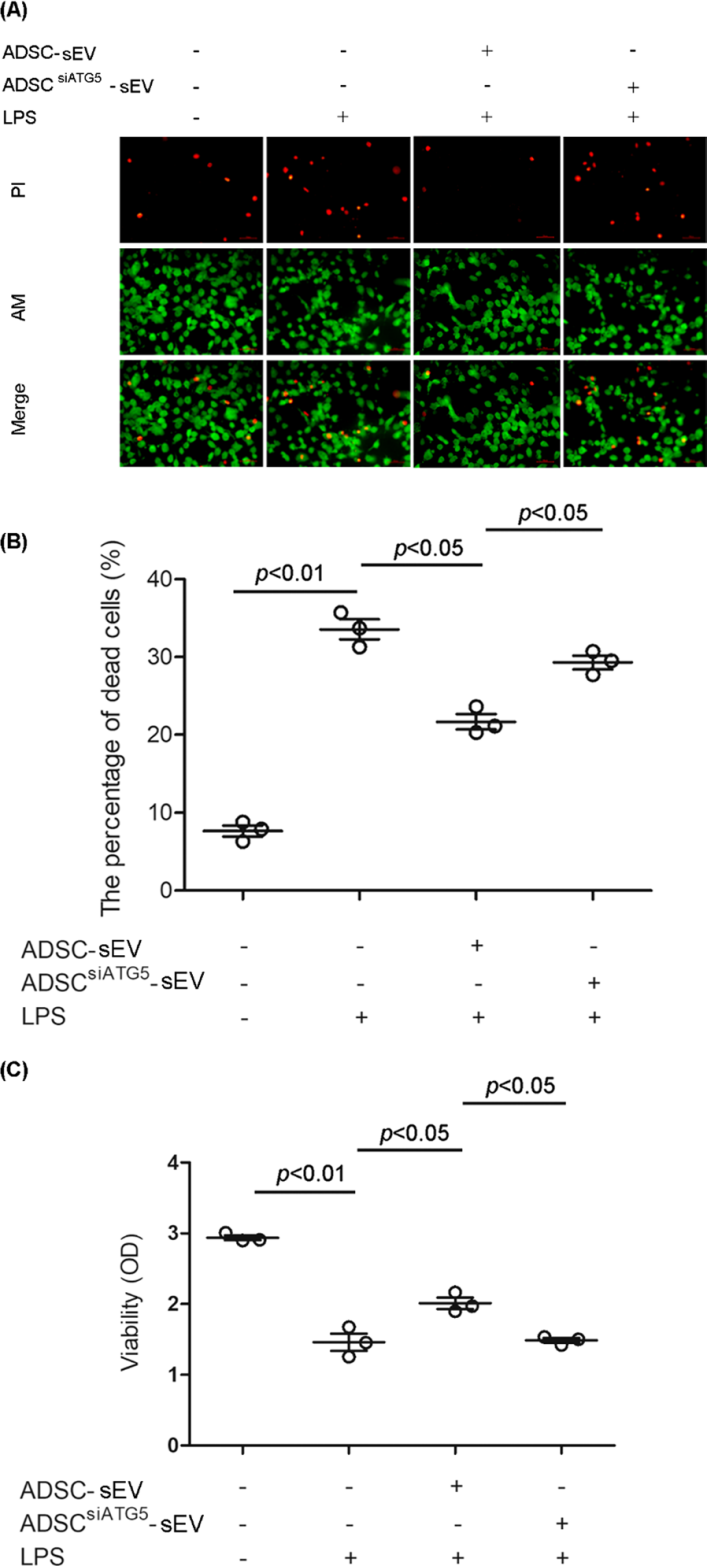
Autophagy mediated the protective effect of ADSC-sEVs on LPS-induced PMVEC viability. **A**, **B** Representative fluorescence images and statistical analysis of LPS-induced endothelial cell death after incubation with sEVs derived from ADSC in the presence or absence of autophagy inhibition. LPS treatment significantly increased the percentage of dead cells, which were characterized by PI staining of the nuclei. Small extracellular vesicle pretreatment markedly alleviated LPS-induced cell death. However, autophagy inhibition markedly weakened sEV-mediated abrogation of cell death [the percentage of dead cells (%), control, 7.67 ± 1.27 vs. LPS, 33.57 ± 2.20 vs. LPS + ADSC-sEV, 21.67 ± 1.72 vs. LPS + ADSC^siATG5^-sEV, 29.30 ± 1.51]. **C** MTT assays assessing the viability of PMVECs after LPS challenge. LPS significantly reduced cell viability, which was apparently alleviated by ADSC-sEVs. Autophagy inhibition reduced the effect of sEVs [the cell viability (OD), control, 2.94 ± 0.06 vs. LPS, 1.46 ± 0.21 vs. LPS + ADSC-sEV, 2.01 ± 0.14 vs. LPS + ADSC^siATG5^-sEV, 1.49 ± 0.06]. The results are expressed as the mean ± SD of three independent experiments

Cell viability was measured to further test the effect of autophagy on sEV function. A LIVE/DEAD viability/cytotoxicity kit was used to investigate cell viability. As shown, LPS treatment significantly increased the percentage of dead cells, which were characterized by PI staining of the nuclei. Small extracellular vesicle pretreatment markedly alleviated LPS-induced cell death. However, autophagy inhibition markedly weakened sEV-mediated abrogation of cell death (Fig. 5A, B).

In addition, an MTT assay was used to further test cell viability. LPS significantly reduced cell viability, which was apparently alleviated by ADSC-sEVs. Autophagy inhibition reduced the effect of sEVs (Fig. 5C).

### Autophagy inhibition reduced the protective effect of ADSC-sEVs on pulmonary microvascular permeability

**Fig. 6:**
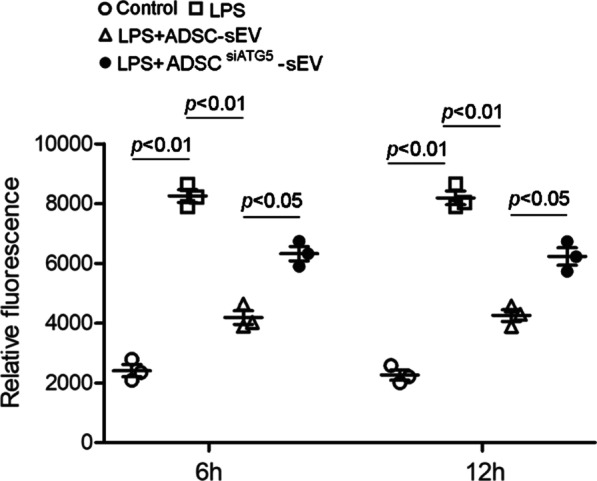
The permeability of PMVECs was measured using a Transwell assay. LPS increased the permeability of endothelial cells, and this effect was significantly alleviated by ADSC-sEVs treatment. Autophagy inhibition weakened the protective effect of ADSC-sEVs against LPS-induced endothelial permeability. (the relative fluorescence at 6 h, control, 2407.67 ± 353.56 vs. LPS, 8251.00 ± 369.21 vs. LPS + ADSC-sEV, 4185.00 ± 396.32 vs. LPS + ADSC^siATG5^-sEV, 6319.67 ± 421.00. the relative fluorescence at 12 h, control, 2260.33 ± 294.18 vs. LPS, 8190.00 ± 397.31, LPS + ADSC-sEV, 4251.00 ± 340.95 vs. LPS + ADSC^siATG5^-sEV, 6228.33 ± 499.55). The experiment was repeated three times

Microvascular permeability has been used as one of the representative indices to assess pulmonary microvascular barrier integrity [24]. In this study, we found that LPS stimulation for 6 h or 12 h increased microvascular endothelial cell permeability, which was significantly reduced by sEV treatment. Autophagy inhibition markedly weakened the effect of ADSC-sEVs on LPS-induced microvascular permeability (Fig. 6).

### Autophagy inhibition reduced the effect of ADSC-sEVs on the LPS-induced formation of stress fibers in PMVECs

**Fig. 7:**
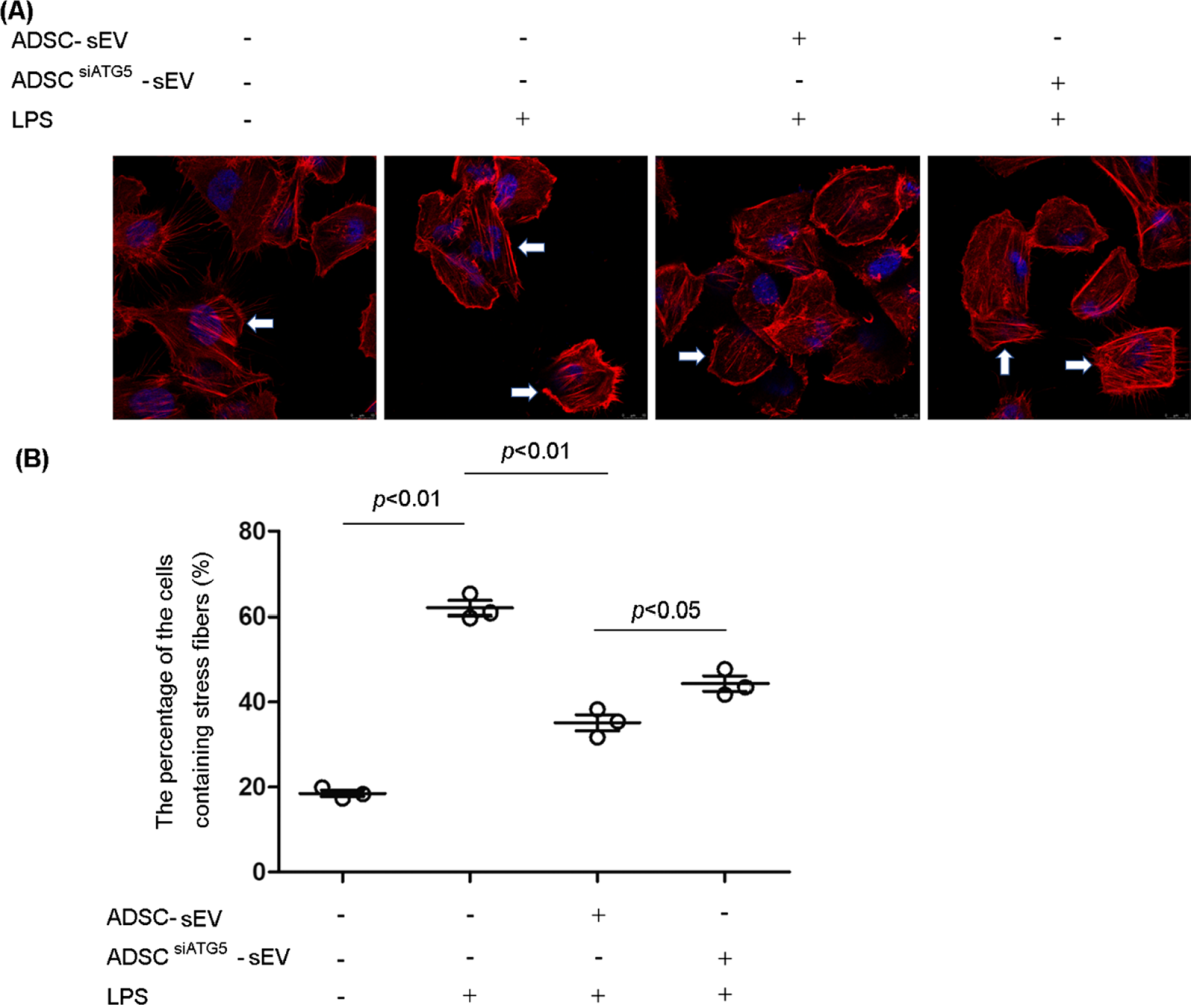
The effect of ADSC-sEVs with or without autophagy inhibition on stress fiber formation. **A** Representative fluorescence images showing stress fibers labeled with F-actin staining. Nuclei were stained with DAPI. LPS significantly increased the formation of actin stress fibers, and this effect was significantly inhibited by sEVs. However, autophagy inhibition reduced the effect of sEVs on stress fiber formation. White arrows indicated the stress fibers labeled with F-actin. **B** Statistical analysis of the percentage of cells containing stress fibers in each experimental group. LPS treatment markedly increased the proportion of cells containing stress fibers, which was effectively decreased by ADSC-sEV treatment. However, autophagy inhibition weakened the effect of ADSC-sEVs on the formation of stress fibers (the percentage of cells containing stress fibers (%), control, 18.53 ± 1.31 vs. LPS, 61.97 ± 2.95 vs. LPS + ADSC-sEV, 35.13 ± 3.31 vs. LPS + ADSC^siATG5^-sEV, 44.37 ± 3.09). The results are expressed as the mean ± SD of three independent experiments

Previous studies have shown that LPS induces F-actin polymerization to form contractile actin bundles and stress fibers. Contraction of stress fibers leads to the formation of intercellular gaps that increase the permeability of the endothelial barrier [25, 26]. To test whether LPS-induced stress fiber formation could be regulated by sEVs, we incubated endothelial cells with sEVs from ADSCs with or without autophagy inhibition under LPS stimulation. As shown, LPS significantly increased the formation of actin stress fibers, and this effect was significantly inhibited by sEVs. However, autophagy inhibition reduced the effect of sEVs on stress fiber formation (Fig. 7A). We further quantified the percentage of cells containing stress fibers in the different groups. LPS treatment markedly increased the proportion of cells containing stress fibers, which was effectively decreased by ADSC-sEV treatment. However, autophagy inhibition weakened the effect of ADSC-sEVs on the formation of stress fibers (Fig. 7B).

### Regulation of autophagy affected the treatment efficiency of ADSC-sEVs in LPS-induced acute lung injury and excessive inflammatory reaction

**Fig. 8:**
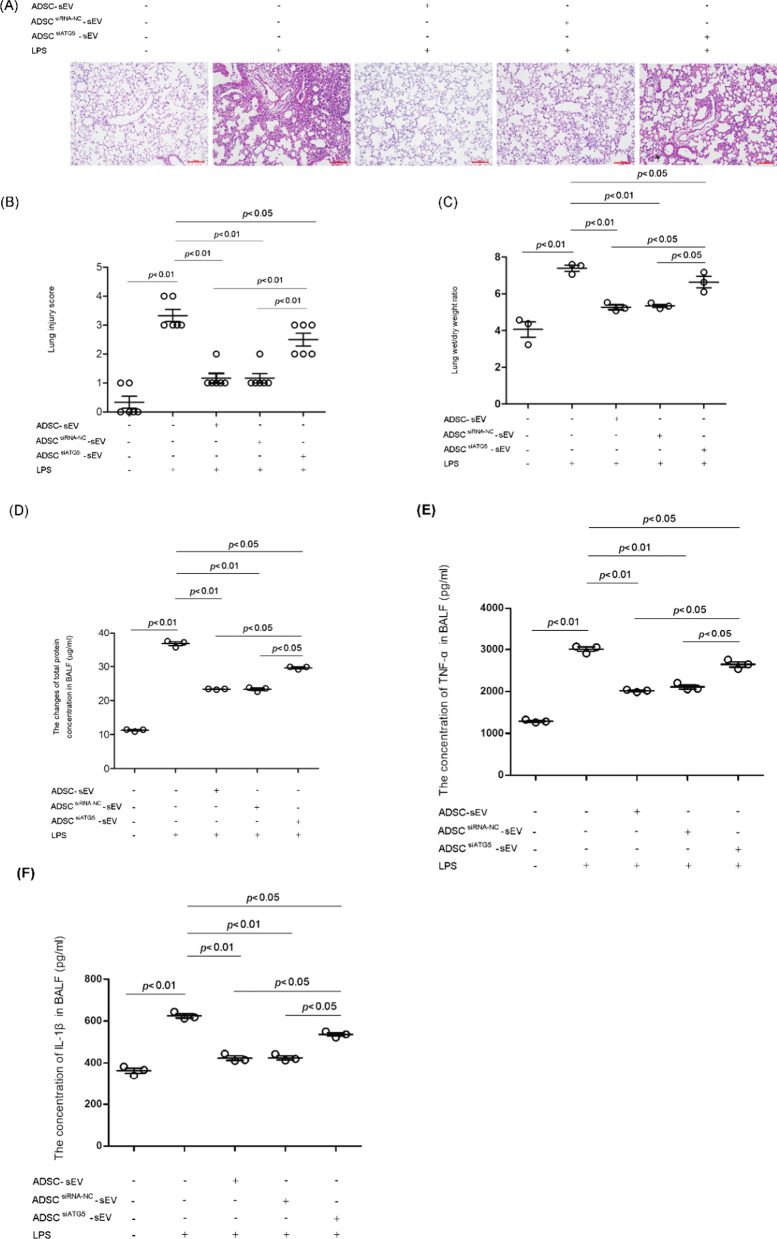
The effect of ADSC-sEVs with or without autophagy inhibition on LPS-induced acute lung injury. **A** Lung histology visualized with hematoxylin and eosin staining. **B** Microscopic injury of the lung was statistically scored. Administration of ADSC-sEV effectively attenuated LPS-triggered lung injury. The effect of ADSC^siRNA−NC^-sEV on lung injury was similar with that of ADSC-sEV. However, the protective effect of ADSC^siATG5^-sEV was much worse than that of ADSC-sEV or ADSC^siRNA−NC^-sEV. The results are presented as the mean ± SD (the lung injury score, control, 0.33 ± 0.52 vs. LPS, 3.33 ± 0.52 vs. LPS + ADSC-sEV, 1.17 ± 0.41 vs. LPS + ADSC^siRNA−NC^-sEV, 1.17 ± 0.41 vs. ADSC^siATG5^-sEV, 2.50 ± 0.55). **C** Lung water content was tested by detecting the wet/dry weight ratio. LPS challenge increased the lung wet/dry weight ratio, which was effectively attenuated by ADSC-sEV treatment. However, the inhibitory action of ADSC^siATG5^-sEV on LPS-induced lung edema was much weaker than that of ADSC-sEVs (the lung wet/dry weight ratio, control, 4.05 ± 0.73 vs. LPS, 7.39 ± 0.29 vs. LPS + ADSC-sEV, 5.27 ± 0.23 vs. LPS + ADSC^siRNA−NC^-sEV, 5.34 ± 0.15. vs. ADSC^siATG5^-sEV, 6.63 ± 0.54). **D** Lung microvascular permeability was assessed by detecting the changes in total protein concentration in bronchoalveolar lavage fluid (BALF). LPS markedly increased protein leakage in BALF, which was significantly attenuated by ADSC-sEV or ADSC^siATG5−NC^-sEV treatment. However, the inhibitory effect of ADSC^siATG5^-sEV on protein leakage in BALF was markedly weaker than that of ADSC-sEV (the total protein concentration in BALF (μg/ml), control, 11.25 ± 0.33 vs. LPS, 36.71 ± 0.94 vs. LPS + ADSC-sEV, 23.34 ± 0.14 vs. LPS + ADSC^siRNA−NC^-sEV, 23.32 ± 0.62. vs. ADSC^siATG5^-sEV, 29.62 ± 0.44). **E**, **F** ELISA assay was used to detect the concentrations of TNF-α and IL-1β in BALF from mouse lungs that underwent LPS treatment. LPS significantly increased the production of TNF-α and IL-1β, which was effectively inhibited by ADSC-sEV or ADSCs^siATG5^-sEV treatment. However, the inhibitory effects of ADSC^siATG5^-sEVs on the production of TNF-α and IL-1β were significantly weaker than those of ADSC- sEVs (the concentration of TNF-α (pg/ml), control, 1291.21 ± 34.34 vs. LPS, 3011.75 ± 91.08 vs. LPS + ADSC-sEV, 2016.01 ± 28.66 vs. LPS + ADSC^siRNA−NC^-sEV, 2107.39 ± 83.85 vs. ADSC^siATG5^-sEV, 2647.53 ± 111.61). The concentration of IL-1β (pg/ml), control, 362.99 ± 22.17 vs. LPS, 624.98 ± 16.92 vs. LPS + ADSC-sEV, 423.24 ± 18.47 vs. LPS + ADSC^siRNA−NC^-sEV, 425.23 ± 16.06 vs. ADSC^siATG5^-sEV, 536.40 ± 14.23). The results are presented as the mean ± SD. The results are presented as the mean ± SD (n = 18/group, 6 for H&E staining and pathological scores; 6 for wet/dry weight ratio; 6 for BALF collection and assessment of total protein level and TNF-α and IL-1β concentration)

LPS stimulated a striking influx of polymorphonuclear leukocytes into the alveolar space, as well as marked congestion and edema in the lung tissue. Administration of ADSC-sEVs effectively attenuated LPS-triggered lung injury. The effect of ADSC^siRNA−NC^-sEVs on lung injury was similar with that of ADSC-sEVs. However, the protective effect of ADSC^siATG5^-sEVs was much worse than that of ADSC-sEVs or ADSC^siRNA−NC^-sEVs (Fig. 8A). The severity of lung injury was also assessed using a semiquantitative histopathology scoring system, ADSC-sEV treatment effectively decreased LPS-induced lung injury. However, inhibition of autophagy, markedly weakened the therapeutic efficacy of ADSC- sEVs (Fig. 8B).

In addition to morphologic evidence, lung edema was assessed by detecting the lung wet/dry weight ratio. LPS challenge increased the lung wet/dry weight ratio, which was effectively attenuated by ADSC-sEV treatment. However, the inhibitory action of ADSC^siATG5^-sEVs on LPS-induced lung edema was much weaker than that of ADSC-sEVs (Fig. 8C).

In vivo lung microvascular permeability was assessed by detecting the total protein concentration in BALF. As shown, LPS markedly increased protein leakage in BALF, which was significantly attenuated by ADSC-sEV or ADSC^siATG5−NC^-sEV treatment. However, the inhibitory effect of ADSC^siATG5^-sEVs on protein leakage in BALF was markedly weaker than that of ADSC-sEVs (Fig. 8D).

An exaggerated inflammatory response is one of the causes of LPS-associated ALI. To evaluate the inflammatory reaction, we measured the levels of TNF-α and IL-1β, two important proinflammatory cytokines, in BALF. As shown, LPS significantly increased the production of TNF-α and IL-1β, which was effectively inhibited by ADSC-sEV or ADSCs^siATG5^-sEV treatment. However, the inhibitory effects of ADSC^siATG5^-sEVs on the production of TNF-α and IL-1β were significantly weaker than those of ADSC- sEVs (Fig. 8E, F).

### Autophagy affected the effect of ADSC-sEVs on toll-like receptor 4 (TLR4)—nuclear factor (NF)-κB signaling in LPS-triggered PMVECs

**Fig. 9:**
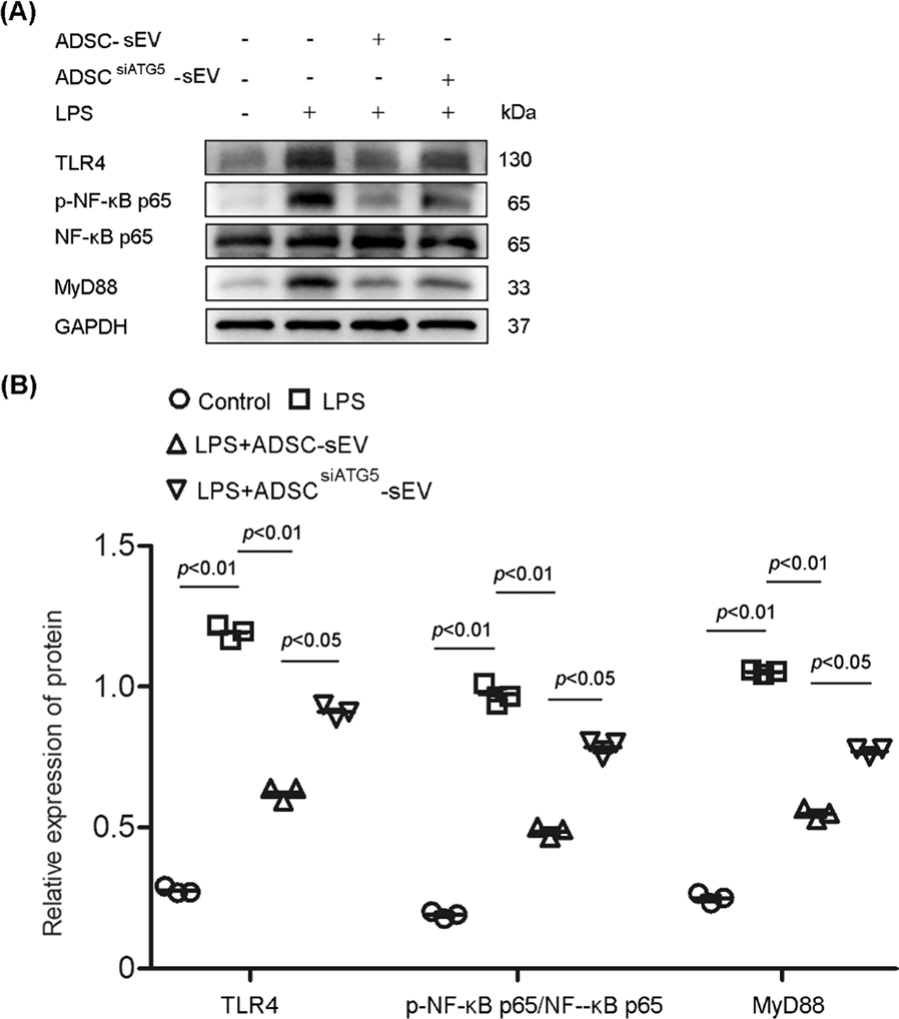
The effect of ADSC-sEVs with or without autophagy inhibition on toll-like receptor 4 (TLR4)-nuclear factor (NF)-κB signaling in LPS-triggered PMVECs. **A** Representative western blots showing expressions of TLR4, MyD88 and total or phosphorylated(p)- NF-κB p65. **B** Statistical analysis of the relative expressions level of TLR4, MyD88 and phosphorylated(p)- NF-κB p65. LPS promoted the expression of TLR4, MyD88 and phosphorylated(p)-NF-κB p65. ADSC-sEVs treatment decreased expressions of all the aforementioned proteins. Autophagy inhibition by siATG5, however, significantly weakened the effect of ADSC-sEVs on inhibiting the TLR4/NF-κB signaling related proteins (the relative expressions level of TLR4, control, 0.28 ± 0.01 vs. LPS, 1.19 ± 0.03 vs. LPS + ADSC-sEV, 0.62 ± 0.03 vs. ADSC^siATG5^-sEV, 0.91 ± 0.02. the relative expressions level of phosphorylated(p)- NF-κB p65, control, 0.19 ± 0.01 vs. LPS 0.97 ± 0.04 vs. LPS + ADSC-sEV, 0.49 ± 0.02 vs. ADSC^siATG5^-sEV, 0.79 ± 0.03. the relative expressions level of MyD88, control, 0.25 ± 0.02 vs. LPS 1.05 ± 0.01 vs. LPS + ADSC-sEV, 0.55 ± 0.02 vs. ADSC^siATG5^-sEV, 0.77 ± 0.02). The results are expressed as the mean ± SD of three independent experiments

TLR4/NF-κB signaling has been verified the crucial role in accelerating the production of proinflammatory cytokines and the development of unbalanced inflammatory response under LPS-induced lung injury. In present study, we determined the effects of ADSC-sEVs with or without autophagy inhibition on TLR4/NF-κB signaling in LPS-triggered PMVECs. As shown, LPS treatment activated TLR4/NF-κB signaling through promoting the expression of TLR4, MyD88 and phosphorylated(p)- NF-κB p65. ADSC-sEVs alleviated the activation of LPS-induced TLR4/NF-κB signaling. Autophagy inhibition by siATG5, however, significantly weakened the effect of ADSC-sEVs on inhibiting the TLR4/NF-κB signaling activation (Fig. 9A and B).

### Autophagy affected the expression of specific miRNAs in ADSC-sEVs

**Fig. 10:**
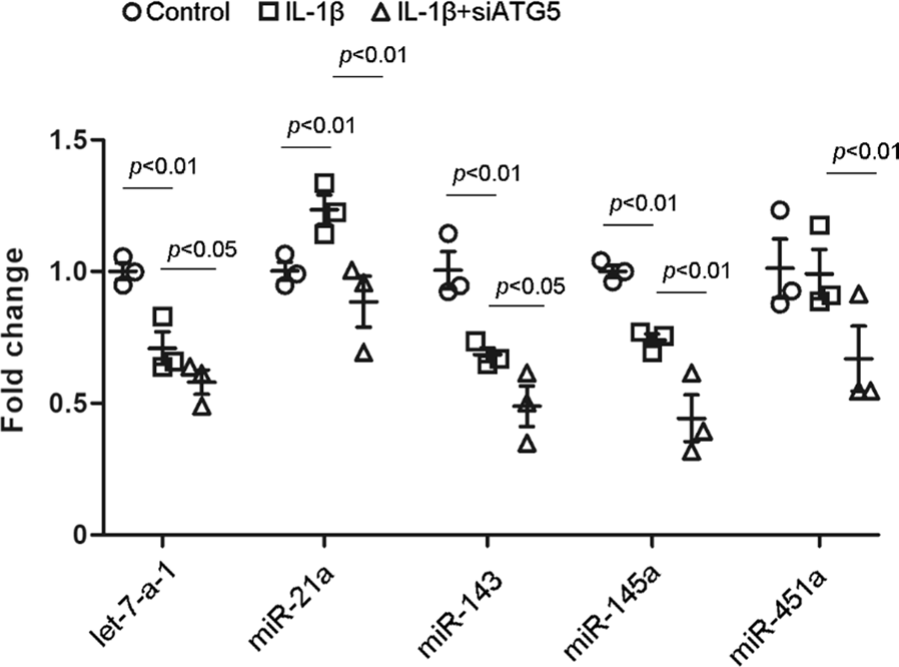
Autophagy mediates the miRNA expression profile of ADSC-sEVs. The expression of let-7-a-1, miR-21a, miR-143, miR-145a and miR-451a in ADSC-sEVs. IL-1β treatment increased the expression of miR-21a and decreased that of let-7-a-1, miR-143 and miR-145a but did not affect the expression of miR-451a. Interestingly, autophagy inhibition weakened the expression of all these miRNAs under IL-1β stimulation. The results are expressed as the mean ± SD of three independent experiments (fold change of let-7-a-1, control, 1.00 ± 0.05 vs. ADSC^IL−1β^-sEV, 0.71 ± 0.10 vs. ADSC^IL−1β+siATG5^-sEV, 0.58 ± 0.08. fold change of miR-21a, control, 1.00 ± 0.06 vs. ADSC^IL−1β^-sEV, 1.23 ± 0.09 vs. ADSC^IL−1β+siATG5^-sEV, 0.89 ± 0.17. fold change of miR-143, control, 1.00 ± 0.12 vs. ADSC^IL−1β^-sEV, 0.69 ± 0.05 vs. ADSC^IL−1β+siATG5^-sEV, 0.49 ± 0.13. fold change of miR-145a, control, 1.00 ± 0.04 vs. ADSC^IL−1β^-sEV, 0.74 ± 0.04 vs. ADSC^IL−1β+siATG5^-sEV, 0.44 ± 0.15. fold change of miR-451a, control, 1.01 ± 0.19 vs. ADSC^IL−1β^-sEV, 0.99 ± 0.16 vs. ADSC^IL−1β+siATG5^-sEV, 0.67 ± 0.21)

To test the effect of autophagy on bioactive components transferred by ADSC-sEVs, we detected the expression changes of specific miRNAs (let-7-a-1, miR-21a, miR-143, miR-145a and miR-451a) that have been found in ADSC-sEVs [27]. We measured the expression profile of the aforementioned miRNAs in ADSC-sEVs with or without autophagy inhibition. IL-1β treatment increased the expression of miR-21a and decreased that of let-7-a-1, miR-143 and miR-145a but did not affect the expression of miR-451a. Interestingly, autophagy inhibition weakened the expression of all these miRNAs under IL-1β stimulation (Fig. 10).

## Discussion

In this study, we found that ADSC-sEVs protect against LPS-induced pulmonary microvascular barrier damage and acute lung injury by alleviating apoptosis and reducing the loss of the tight junction-related proteins ZO-1 and claudin-5. Autophagy is one of the essential regulators of the protective effect of ADSC-sEVs by affecting the expression profiles of at least the aforementioned five specific miRNAs within ADSC-sEVs.

sEVs are one of the pivotal components of stem cell paracrine signaling and have been shown to be much more effective in some organ injuries and repairs than direct stem cell differentiation. Under normal conditions, most cells can secrete sEVs; however, pathogens or other stress stimuli may promote sEV secretion and/or alter sEVs contents [28–30]. Hypoxic preconditioning enhanced the protective effect of bone marrow stromal cell-derived exosomes against acute myocardial infarction [31]. Ischemic preconditioning can potentiate the protective effect of marrow stromal cell-derived exosomes on endotoxin-induced acute lung injury [32]. In addition, LPS pretreatment not only induced exosome secretion by macrophages but also enhanced the effects of macrophage-derived exosomes on the proliferation and activation of hepatic stellate cells [33]. Similarly, in the present study, we showed that preconditioning with IL-1β, one of the key proinflammatory factors induced by LPS, promoted the production of ADSC-sEVs and affected the expression of miRNAs in sEVs. Based on these findings, IL-1β preconditioning is a viable option to enhance sEV functions and prevent LPS-induced lung injury.

In the present study, we found that sEV treatment significantly reduced endothelial cell apoptosis, which is one of the classic characteristics of LPS-induced endothelial barrier damage. Our findings are consistent with those of previous studies. The administration of exosomes to staurosporine-treated Chinese hamster ovary cells effectively alleviated apoptosis and enhanced cellular viability [34]. In a skin lesion model, ADSC-Exos inhibited HaCaT cell apoptosis and promoted cell proliferation to accelerate cutaneous wound healing [35]. However, sEVs released from different cell types have different biological effects, and different stress stimuli may trigger different functions in homologous sEVs. Some researchers have found that tumor-derived exosomes carrying immunosuppressive factors can induce apoptosis in activated CD8 + cells and NK cells to suppress immunotherapy efficacy [36]. These findings suggest that the effects of sEVs on target cell apoptosis are not uniform and that the sEV origin and pathological conditions may be key regulatory factors.

The bioactivity of sEVs is ultimately attributed to their protein and nucleic acid components. miRNAs are the most numerous cargo molecules in sEVs; they are selectively sorted into sEVs and transferred to recipient cells, where they mediate certain target mRNAs and cell functions. In the present study, we found that stimulation with IL-1β increased the expression of miR-21a and decreased that of let-7-a-1, miR-143 and miR-145a, but did not affect the expression of miR-451a. These findings suggest that more than one miRNA participates in regulating the effects of sEVs in alleviating LPS-induced endothelial barrier damage. Although many previous studies have highlighted the pivotal effects of sEV miRNAs, many of these studies focused on the function of specific miRNAs. Our findings suggest that sEV functions are likely to be due to the cooperative effects of various miRNAs. Aforementioned miRNAs have been testified the different roles in some inflammation condition. For instance, miR-21 has been shown the anti-apoptosis and anti-inflammatory effect through downregulating expressions of some proapoptotic and proinflammatory genes [37]. miR-let-7 overexpression suppressed pro-inflammatory cytokine production in α-Syn-induced Parkinson’s mice [38]. However, miR-145a expression is positively correlated with inflammation in some pathological condition [39]. It is essential for us to implement further studies to verify the exact role of each miRNA aforementioned and clarify the relevance and crosstalk among them under LPS-triggered inflammatory conditions.

Autophagy, which is a lysosomal-dependent degradation and recycling pathway, has traditionally been suggested to maintain protein, lipid and organelle homeostasis. Recently, autophagy has been identified as one of the vital mediators of sEV biogenesis and function. We found that the same concentration of sEVs collected from ADSCs in the presence or absence of autophagy inhibition had different protective effects on LPS-induced pulmonary microvascular endothelial barrier damage and lung injury. Autophagy inhibition partially weakened the protective effect of ADSC-sEVs, as indicated by increases in the apoptosis rate and stress fiber formation but reduced expression of tight junction-related proteins in endothelial cells. These data provide extremely strong evidence suggesting that autophagy can affect sEV functions. Our findings are consistent with those of previous studies. Autophagy regulation modulates the effect of retinal astrocyte‐derived exosomes on the proliferation and migration of endothelial cells [40]. On the one hand, autophagy shares molecular machinery with exosome biogenesis, and there is substantial crosstalk between these two processes [10]. In addition to its traditional roles in maintaining protein, lipid and organelle homeostasis, increasing evidence indicates that autophagy can impact RNA homeostasis. Beyond its other degradative capabilities, autophagy can degrade RNA, RNA-binding proteins and ribonucleoprotein complexes [13, 41]. In the present study, we found that autophagy inhibition lowered the expression of let-7-a-1, miR-21a, miR-143, miR-145a and miR-451a under IL-1β stimulation in ADSC-sEVs. This result likely explains why autophagy affects sEV functions. Further studies are essential to classify the mechanism by which autophagy mediates sEV miRNA expression.

## Conclusion

In conclusion, we showed that ADSC-sEVs were beneficial in maintaining pulmonary microvascular barrier integrity and lung injury. Autophagy inhibition affected the expression levels of let-7-a-1, miR-21a, miR-143, miR-145a and miR-451a and mediated the protective effects of ADSC-sEVs against LPS-induced damage to the lung microvascular endothelial barrier. These results provide new insights into the roles and the related mechanisms of ADSC-sEVs in LPS-induced acute lung injury and suggest that autophagy regulation might be a potential strategy for modulating the treatment efficacy of ADSC-sEVs in lung injury.

## Supplementary Information

**Additional file 1.** Supplementary figures.

## Data Availability

The datasets generated/analyzed during the current study are available.

## Abbreviations

LPS: Lipopolysaccharide
ADSC: Adipose-derived stem cell
ADSC-sEVs: ADSC-derived small extracellular vesicles
miRNA: MicroRNA
siATG5: Small-interfering RNA targeting autophagy-related gene 5
ALI: Acute lung injury
ZO-1: Zonula occludens-1
PMVECs: Pulmonary microvascular endothelial cells
