# Supplementary material for: Autophagy regulates the effects of ADSC-derived small extracellular vesicles on acute lung injury
Source: Respir Res. 2022 Jun 9;23:151. doi: 10.1186/s12931-022-02073-y (PMC9185906; doi:10.1186/s12931-022-02073-y)
Supplement: Supplementary file 1 — Additional file 1. Supplementary figures. [file 12931_2022_2073_MOESM1_ESM.docx]

**Supplementary figures**


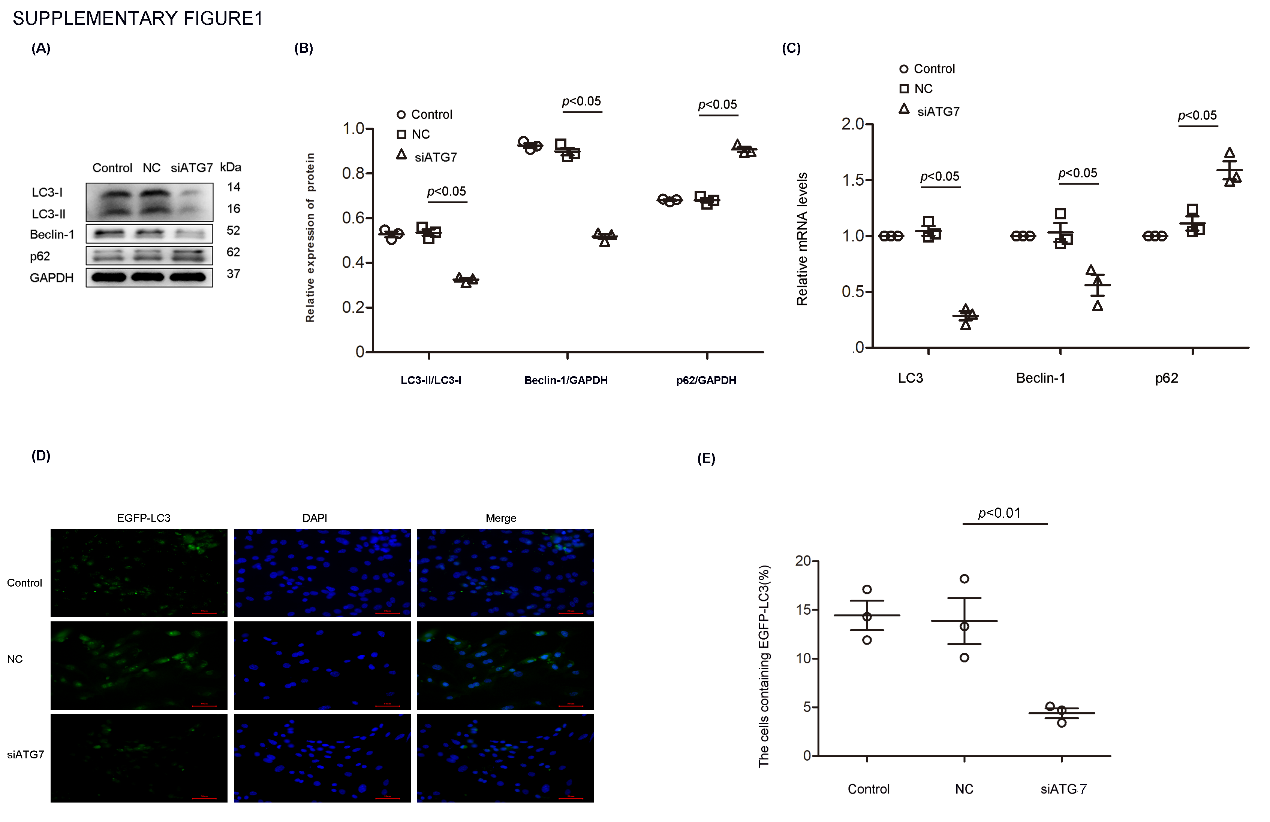
**Supplementary Figure. 1 Effects of siATG7 on autophagy in ADSCs.** (A) Representative western blots showing expression of LC3, Beclin-1 and p62 in ADSCs. (B) Statistical analysis of LC3, Beclin-1 and p62 expression after treatment with siATG7. siATG7 treatment markedly inhibited expression of LC3-II and Beclin-1 but promoted that of p62. (C) Relative mRNA level of LC3, Beclin-1 and p62. siATG7 treatment decreased the mRNA level of LC3 and Beclin-1 but increased that of p62. (D) Fluorescent images of EGFP-LC3 in ADSCs. （E）Statistical analysis of the number of cells containing LC3-positive puncta in different groups. The number of cells containing LC3-positive puncta in LPS treatment group was much greater than that in control group, and the number of puncta was decreased by siATG7 treatment. The results are expressed as the mean ± SD of three independent experiments.


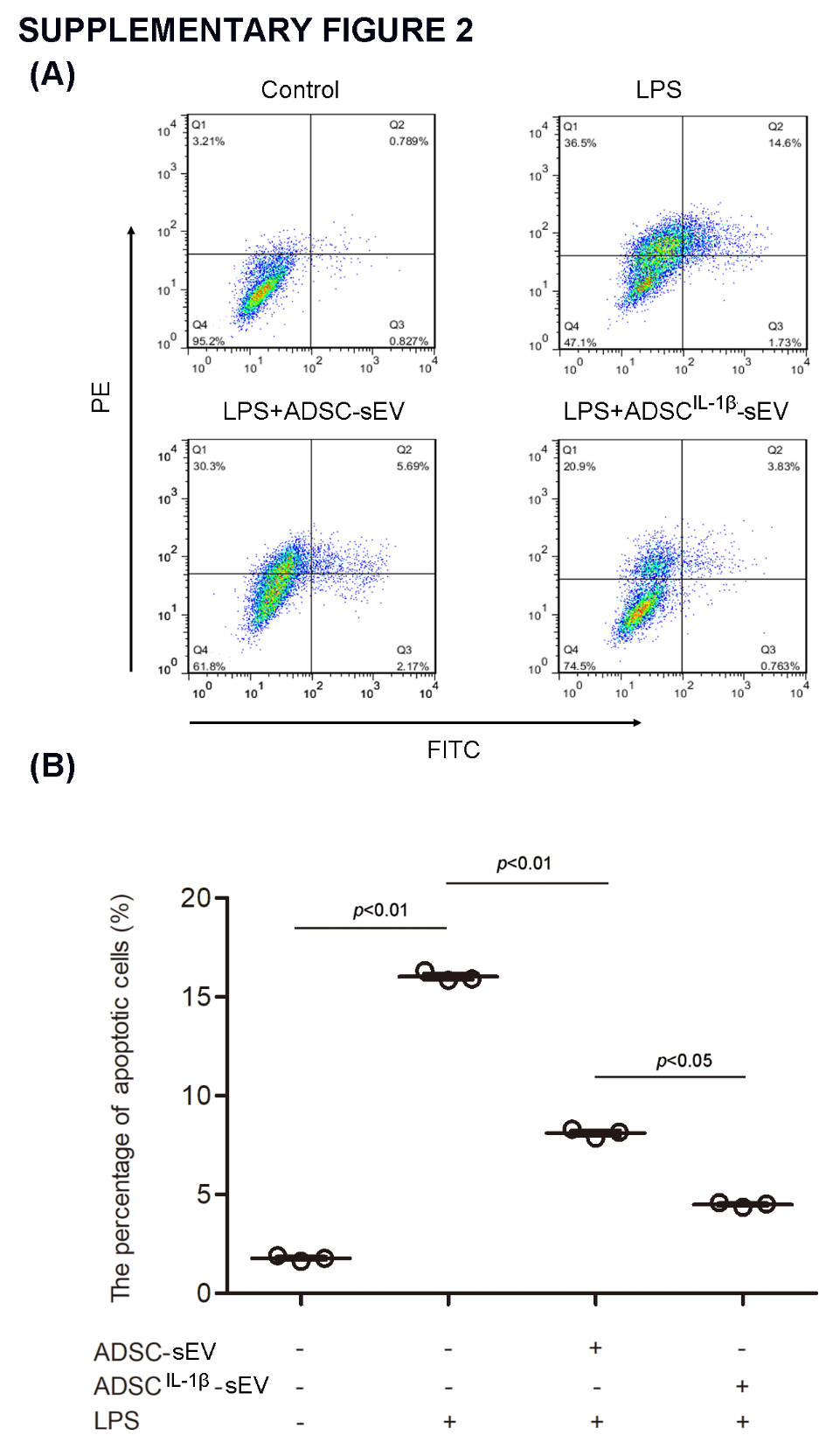


**Supplementary Figure 2. IL-1β preconditioning affected the effect of ADSC-sEVs** **on LPS-****induced PMVEC apoptosis**. (A) Typical flow cytometry quadrant diagrams for assessing apoptotic PMVECs. The top left, top right, and bottom right plots represent necrotic cells and late and early apoptotic cells, respectively. (B) Statistical analysis of PMVEC apoptosis. LPS markedly increased the percentage of endothelial cell apoptosis, which was effectively reduced by ADSC- sEVs. IL-1β preconditioning enhanced the protective effect of ADSC- sEVs against LPS-induced PMVEC apoptosis. The results are expressed as the mean ± SD of three independent experiments.


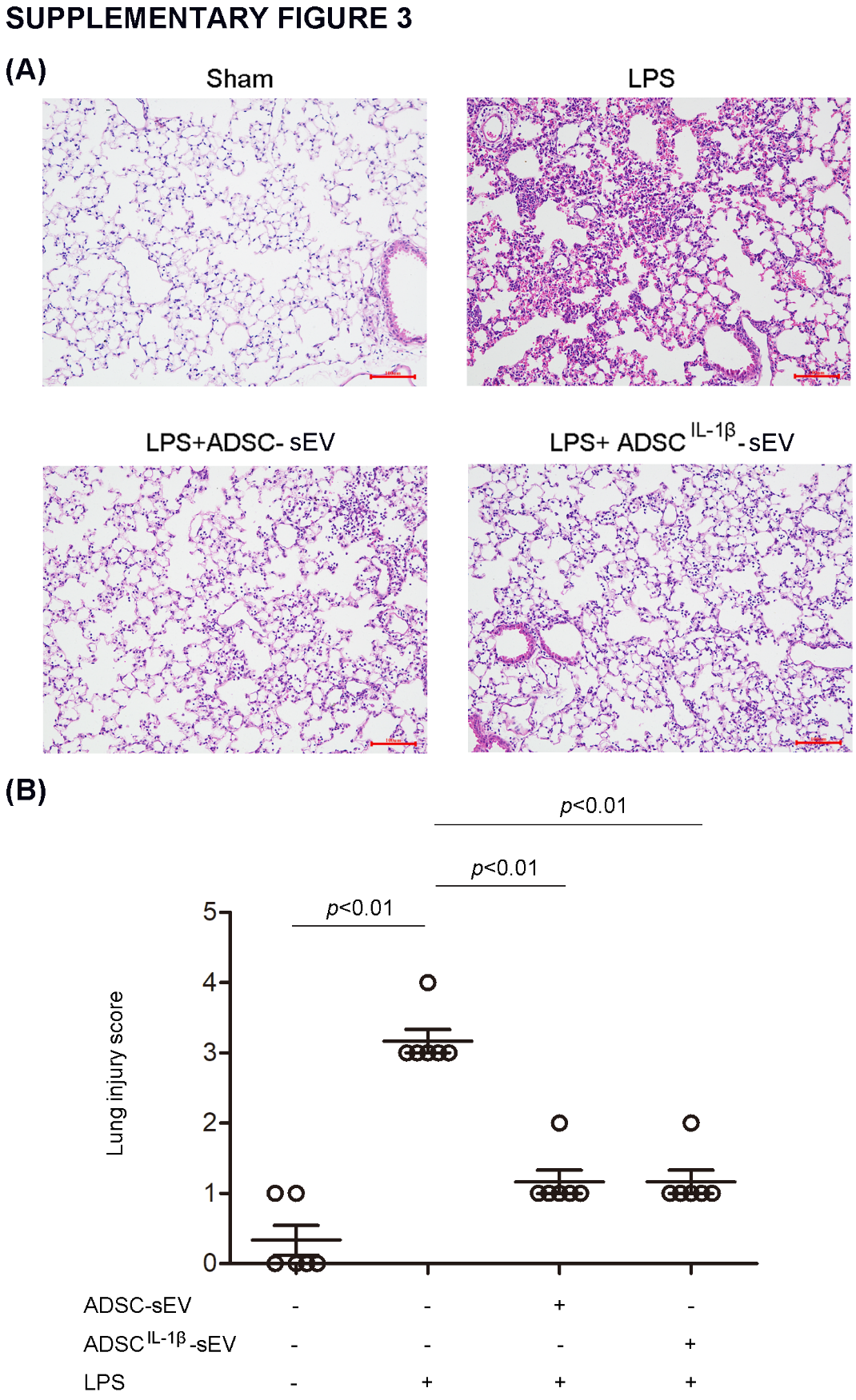


**Supplementary Figure 3. IL-1β preconditioning affected the effect of ADSC-sEVs** **on LPS-induced acute lung injury**. (A) Lung histology visualized with hematoxylin and eosin staining. (B) Microscopic injury of the lung was statistically scored. Administration of ADSC-sEVs effectively attenuated LPS-triggered lung injury. The effect of ADSC^IL-1β^-sEVs on lung injury was similar with that of ADSC-sEVs. The results are presented as the mean ± SD.


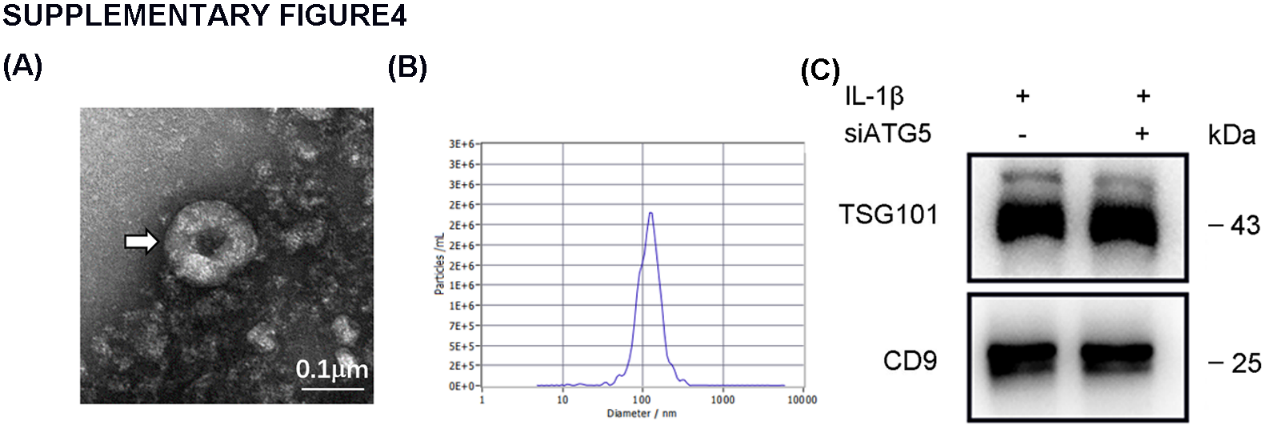


**Supplementary Figure 4.** **Assessment the effect of siATG5 on ADSC-sEVs biological characteristics.** (A) Electron microscopy showing the cup-shaped morphology of sEVs in siATG5-treated ADSCs. (B) Nanoparticle tracking analysis of sEVs shows a single peak at 100 nm. (C) Representative western blot analysis of the expressions of TSG 101 and CD9 in ADSC-sEVs in presence or absence of siATG5 treatment.


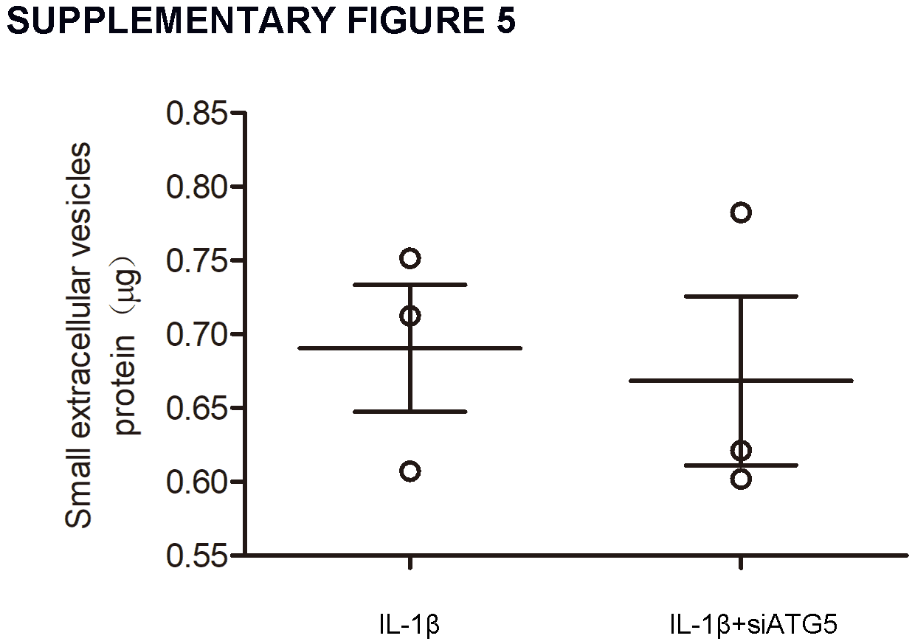


**Supplementary Figure 5. Assessment of the quantitative consistency** **in ADSC-sEVs between groups.** Quantitative analysis of total protein in ADSC-sEVs in presence or absence of siATG5 treatment. There were not significant differences in the total amount of protein between ADSC-sEVs and ADSC^siATG5^-sEVs. The results are expressed as the mean ± SD of three independent experiments.


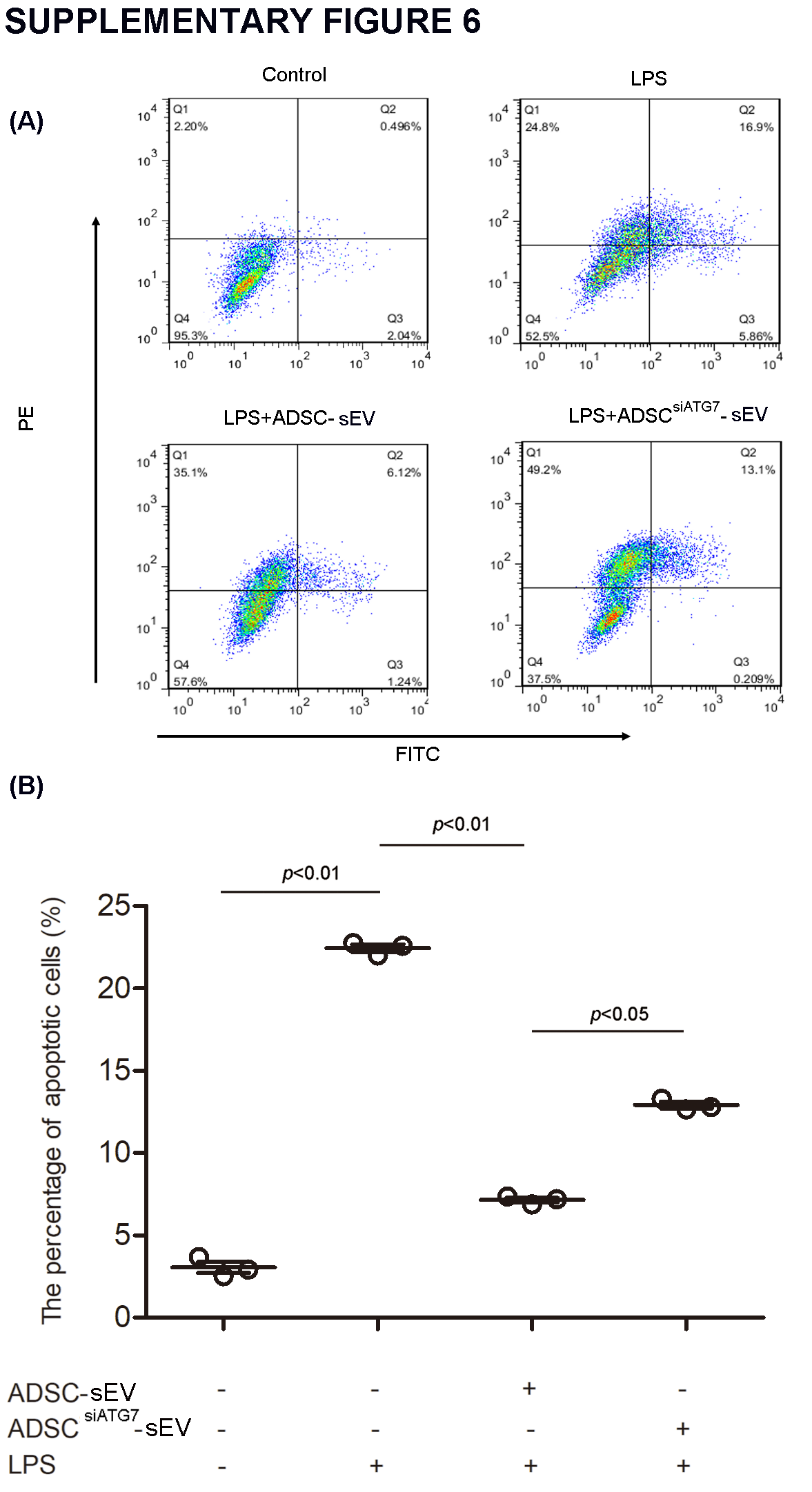


**Supplementary Figure 6. Autophagy inhibition by siATG7 weakened the inhibitory effect of ADSC-sEVs** **on LPS-induced PMVEC apoptosis**. (A) Typical flow cytometry quadrant diagrams for assessing apoptotic PMVECs. The top left, top right, and bottom right plots represent necrotic cells and late and early apoptotic cells, respectively. (B) Statistical analysis of PMVEC apoptosis. LPS markedly increased the percentage of endothelial cell apoptosis, which was effectively reduced by ADSC- sEVs. siATG7 treatment suppressed the protective effect of ADSC- sEVs against LPS-induced PMVEC apoptosis. The results are expressed as the mean ± SD of three independent experiments.
